# Modeling and characterization of pure and odorant mixture processing in the *Drosophila* mushroom body calyx

**DOI:** 10.3389/fphys.2024.1410946

**Published:** 2024-10-16

**Authors:** Aurel A. Lazar, Tingkai Liu, Chung-Heng Yeh, Yiyin Zhou

**Affiliations:** ^1^ Bionet Group, Department of Electrical Engineering, Columbia University, New York, NY, United States; ^2^ Cold Spring Harbor Laboratory, Cold Spring Harbor, NY, United States; ^3^ Department of Computer and Information Science, Fordham University, New York, NY, United States

**Keywords:** *Drosophila*, olfaction, divisive normalization, calyx, APL, kenyon cell, odorant mixtures, pure odorants

## Abstract

Associative memory in the Mushroom Body of the fruit fly brain depends on the encoding and processing of odorants in the first three stages of the Early Olfactory System: the Antenna, the Antennal Lobe and the Mushroom Body Calyx. The Kenyon Cells (KCs) of the Calyx provide the Mushroom Body compartments the identity of pure and odorant mixtures encoded as a train of spikes. Characterizing the code underlying the KC spike trains is a major challenge in neuroscience. To address this challenge we start by explicitly modeling the space of odorants using constructs of both semantic and syntactic information. Odorant semantics concerns the identity of odorants while odorant syntactics pertains to their concentration amplitude. These odorant attributes are multiplicatively coupled in the process of olfactory transduction. A key question that early olfactory systems must address is how to disentangle the odorant semantic information from the odorant syntactic information. To address the untanglement we devised an Odorant Encoding Machine (OEM) modeling the first three stages of early olfactory processing in the fruit fly brain. Each processing stage is modeled by Divisive Normalization Processors (DNPs). DNPs are spatio-temporal models of canonical computation of brain circuits. The end-to-end OEM is constructed as cascaded DNPs. By extensively modeling and characterizing the processing of pure and odorant mixtures in the Calyx, we seek to answer the question of its functional significance. We demonstrate that the DNP circuits in the OEM combinedly reduce the variability of the Calyx response to odorant concentration, thereby separating odorant semantic information from syntactic information. We then advance a code, called first spike sequence code, that the KCs make available at the output of the Calyx. We show that the semantics of odorants can be represented by this code in the spike domain and is ready for easy memory access in the Mushroom Body compartments.

## 1 Introduction

Odor signal processing in the olfactory system of diverse organisms is the result of millennia of convergent evolution (Ache and Young, 2005). Unlike other sensory systems such as the visual system, the odor processing pathways are not embedded within topographically organized circuits (such as retinotopy in visual systems (Sanes and Zipursky, 2010)). Instead, olfactory circuits are organized non-topographically (Mombaerts, 1999; Buck, 2005; Shepherd, 2004; Cleland and Sethupathy, 2006), and their affinities to given odorant molecules directly encode the identities of the said stimuli (Buck and Axel, 1991; Firestein, 2001). This unique sensory characterization of the olfactory stimulus space also led to a highly efficient odor signal processing neural circuit.

In *Drosophila Melanogaster*, more than 40% of the total neural real estate is dedicated to processing visual signals (Barish and Volkan, 2015), while about 5% is dedicated to processing olfactory inputs (Scheffer et al., 2020; Masse et al., 2009; Zheng et al., 2017). Neverthless, *Drosophila* have remarkable olfactory-based foraging, mating, and predator avoidance (Vosshall and Stocker, 2007) capabilities. Given the rich olfactory-related behavior repertoire of *Drosophila* (Benton, 2022), its well-mapped olfactory neural circuit and powerful genetic tools, its olfactory system serves as the ideal platform for unraveling the mysteries of olfactory processing.

**FIGURE 1:**
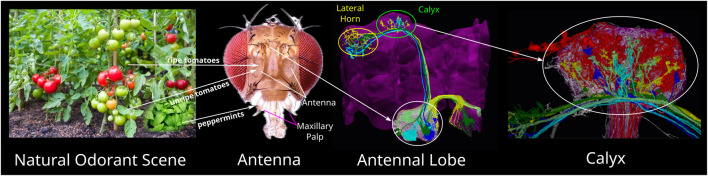
Odorant mixture processing pathways of the Early Olfactory System of the fruit fly. A natural odorant scene (left) may consist of many odorant mixtures, including ripe and unripe tomatoes, and peppermints that repel flies. Odorant mixtures are sensed by the Olfaction Sensory Neurons (OSNs) whose dendrites are located on the Antenna and Maxillary Pulps (second to left, adapted from Fabian and Sachse (2023), under Creative Commons Attribution License (CC-BY)). OSNs project their axons into the Antennal Lobe (AL) (white ellipse in third to left). AL is innervated by a large number of Local Neurons (LNs, white transparent). Projection Neurons (PNs) (colored neurons), the outputs of the AL, send their axons to the Calyx (green ellipse in third to left) and the Lateral Horn (yellow ellipse in third to left). In Calyx (right most), PNs (colors other than red and white) provide inputs into Kenyon Cells (KCs) (red). The Calyx is also innervated by the APL neuron (white transparent) that interacts with the KCs. For interactive 3D visualization of the connectome of the AL and Calyx, see NeuroNLP (2024a) and NeuroNLP (2024b).

In the fruit fly, natural odorant scenes (see Figure 1 first column) are first sensed in the Antenna and Maxillary Palps by the dendrites of thousands of Olfactory Sensory Neurons (OSNs), each expressing a single olfactory receptor (OR) type (Vosshall, 2000) (see Figure 1 second column). The second layer of olfactory sensory processing is the Antennal Lobe (AL, see Figure 1 third column). OSNs expressing the same OR type typically project their axons into a single glomerulus, a dense connectivity region in the AL. The dendritic trees of Projection Neurons (PNs) typically also innervate a single glomerulus. A large number of Local Neurons (LNs) shape the I/O of the AL circuit (Lazar et al., 2022). PNs project their axons to the Mushroom Body Calyx and/or the Lateral Horn (see Figure 1 third column top and fourth column). In the Calyx, some 50 types of PNs synapse onto 2,000 Kenyon Cells (KCs), a rapid expansion of the number of neurons (Modi et al., 2020). A key circuit element in the Calyx is the giant Anterior Paired Lateral (APL) feedback neuron receiving input from all KCs. The third and fourth columns in Figure 1 can also be viewed online as interactive 3D visualizations provided by the Fruit Fly Brain Observatory (Ukani et al., 2019; 2024). The URLs can be found in NeuroNLP (2024a) and NeuroNLP (2024b).

There has been an extensive amount of work in discerning the odorant identity and concentration in the olfactory system of the fruit flies, other insects and vertebrates. It has been shown that odorants typically retain their perceptual identities over a range of concentrations (Blazing and Franks, 2020). In *Drosophila*, the same odorant may recall the memory associated with the odorant over more than an order of magnitude of concentration amplitude values (Masek and Heisenberg, 2008).

Concentration-invariant representation of odorant identity has been proposed at almost every stage of the olfactory circuit, in the Antenna (Egea-Weiss et al., 2018), in the Antennal Lobe (or Olfactory Bulb in vertebrates) (Stopfer et al., 2003; Wilson et al., 2017; Chong et al., 2020; Lazar et al., 2023) and at the KC level of the Mushroom Body (or Piriform Cortex in mammals) (Stopfer et al., 2003; Bolding and Franks, 2018). These studies assumed, however, that the odorant identity is known. In other words, odorant identity has been viewed akin to labels used in supervised learning. A major goal has been to record from the neural activity arising at different stages of the Early Olfactory System and to examine when the recorded signal can be used to increase the accuracy of identifying or classifying odorants (Egea-Weiss et al., 2018; Jeanne and Wilson, 2015; Stopfer et al., 2003). These approaches do not reveal, however, the functional logic of the underlying neural circuits.

**FIGURE 2:**
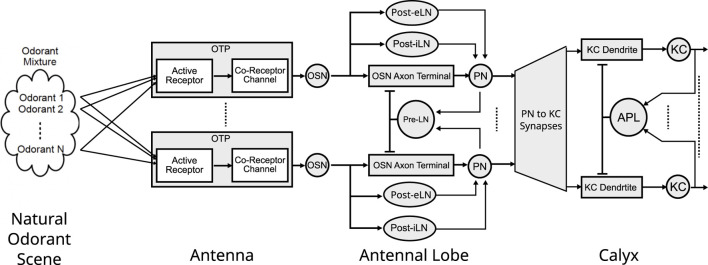
The architecture of the Odorant Encoding Machine (OEM) modeling the early olfactory system of the fruit fly. The OEM consists of a cascade of spatio-temporal Divisive Normalization Processors (DNPs) each modeling the Antenna, Antennal Lobe and Calyx. All odorants in a mixture are sensed and encoded by a molecular Odorant Transduction Process (OTP) of each OSN type. Each OSN type then provides input to an AL channel (glomerulus) with a Projection Neuron (PN) as channel output. Three types of local neurons, Presynaptic Local Neurons (Pre-LNs), Postsynaptic excitatory LNs (Post-eLNs) and Postsynaptic inhibitory LNs (Post-iLNs) are modeled as 3 types of differential DNPs. The Calyx features an expansion of the PN to KC connectivity, as well as a DNPs circuit consisting of the KC dendrites, KC biological spike generators and the APL spatio-temporal feedback neuron.

In previous work, we proposed computational models for mono-molecular odorant encoding and processing in both the Antenna (Lazar and Yeh, 2020) and the Antennal Lobe (Lazar et al., 2023). We advanced a model of olfactory objects of the odorant space that explicitly describes both their identity (odorant semantics) and their concentration amplitude (odorant syntax). Our model of the Antenna then encodes a multiplicatively-coupled representation of the semantic and syntactic information streams, resulting in a confounding representation that is disentangled by the inhibitory and excitatory Local Neurons of the Antennal Lobe. Both models of the Antenna and the Antennal Lobe reproduce with a very high precision the experimentally obtained physiological responses of the Olfactory Sensory Neurons (output neurons of the Antenna) and Projection Neurons (output neurons of the Antennal Lobe) (Kim et al., 2011; 2015). Importantly, by developing a model of the Antennal Lobe that recovers the odorant identity information from the confounding representation of the Antenna, we showed that the functional significance of the Antennal Lobe (in particular its highly diverse inhibitory Local Neurons) is to separate the odorant semantics from syntax, thereby undoing the multiplicatively coupled odorant encoding in the Antenna (see also Figure 2).

To emphasize, the novelty of our approach rests on explicitly modeling the space of odorants using constructs of both semantic and syntactic information, a subtle but profound distinction from the existing literature that solely invokes methods of traditional syntactic information processing. These prior works focused on methods of processing odorant concentration, with odorant identity mentioned in passing and/or lacking computational or theoretical rigor. However, when it comes to understanding the functional logic of olfactory circuits, processing odorant concentration alone turns out to be, as we argue here and elsewhere, a major limitation. Our present work extends the I/O modeling and characterization of semantic/syntactic information processing that we obtained for the Antenna and Antennal Lobe to the MB Calyx circuit. We show how the Calyx extracts and represents semantic information in the spike domain.

The first three layers of the Early Olfactory System depicted in Figure 1 are modeled as the Odorant Encoding Machine (OEM) shown in Figure 2 (Lazar et al., 2020a). The architecture of the OEM consists of three cascaded Divisive Normalization Processors (DNPs), a spatio-temporal extension of the static divisive normalization model previously analyzed by (Olsen and Wilson, 2008; Carandini and Heeger, 2012; Lazar et al., 2020b; Lazar and Zhou, 2023). Note that in vision, it has been recently shown that the motion detection pathway of the early visual system of the fruit fly can also be modeled as a cascade of DNPs (Lazar et al., 2020b; Lazar and Zhou, 2023), suggesting that DNPs as building blocks of computation can be combined to realize more complex processing in the fruit fly brain.

In the current work, we seek to answer the question regarding the functional significance of the Mushroom Body Calyx, the last building block of the OEM cascade shown in Figure 2. Note that in the MB Calyx most of biological real estate is devoted for re-representing odorant identities - with, on average, 40 Kenyon Cells in the Calyx for each 1 Projection Neuron type in the Antennal Lobe. While previous studies have explored the Calyx’s role in associative learning (e.g., Heisenberg, 2003), our focus shall be on modeling the pre-associative representation of odorant identity and exploring how semantics of pure and odorant mixtures are coded for memory access by the Mushroom Body. By (i) abstracting the structural connectome datasets into executable circuit diagrams, and by (ii) focusing on the exploration of the functional logic of the underlying circuits, we follow here the workflow established in Lazar et al. (2021).

This paper is organized as follows. In Section 2, we present the architecture of the OEM. We review the model of the space of odorants and the input/output (I/O) model of the Antenna and Antennal Lobe. The model of the Calyx is detailed next. In Section 3, we extensively characterize and evaluate the processing of pure and odorant mixtures in the Calyx. The KC generated spike train at the output of the Calyx, called the first spike sequence code, represents the odorant identity made available to the Mushroom Body memory circuit. In Section 4 we conclude with a brief discussion.

## 2 Odorant Encoding Machine

A schematic diagram of the Odorant Encoding Machine (OEM) is shown in Figure 2. In what follows we describe the four cascaded building blocks of the OEM, respectively, modeling the space of odorants (see section 2.1), the molecular encoding of mono-molecular odorants and odorant mixtures in the antenna (see section 2.2), the I/O modeling of the antennal lobe (see section 2.3) and the I/O modeling of mushroom body calyx (see section 2.4).

### 2.1 Modeling the space of odorants

**FIGURE 3:**
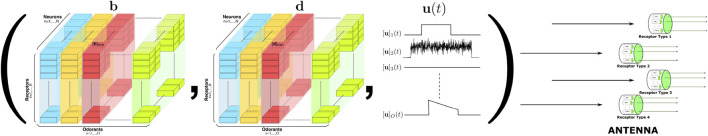
Modeling the space of mono-molecular odorants. Elements of the odorant space are defined by the tensor of odorant-receptor binding rate, dissociation rate and concentration amplitude (b,d,u(t)) . For a given neuron n=1,2,…,N , the binding rate and dissociation rate values are, respectively, denoted by ${\left[b\right]}_{\mathrm{ron}}$ and ${\left[d\right]}_{\mathrm{ron}}$ , for all r=1,2,…,R , and o=1,2,…,O . Single and/or odorant mixtures interact with the receptors expressed by the Olfactory Sensory Neurons in the Antenna (right). Adapted from Lazar et al. (2022), under Creative Commons Attribution License (CC-BY).

The space of mono-molecular odorants (see also Figure 2, left) was first formally modeled and biologically validated in Lazar and Yeh (2020). In this model, the Odorant Transduction Process (OTP) taking place in the cilia of the Olfactory Sensory Neurons (OSNs) (see also Section 2.2) encodes odorants as objects defined by the tensor of binding rates, dissociation rates and concentration amplitude $\left(b,d,u\left(t\right)\right)$ . Tensors are multidimensional arrays that generalize the concept of vectors (1-dimensional arrays) and matrices (2-dimensional arrays). They provide a complex representation of complex data. Here b and d are 3-dimensional tensors (see Figure 3), with each of the three dimensions representing O odorants, R receptors and N OSNs expressing a receptor. Each entry ${\left[b\right]}_{ron},{\left[d\right]}_{ron}$ describes the binding/dissociation rates for n -th OSN expressing receptor r to/from odorant o , n∈1,…,N , r∈1,…,R and o∈1,…,O . The entry ${\left[u\right]}_{o}\left(t\right)$ is the concentration waveform of odorant o , o∈1,…,O (see also Figure 3).

With this odorant object model, the *semantics* of the space of mono-molecular odorants (Lazar et al., 2023) is defined by the 2-tuple of binding/dissociation rate tensors (b,d) , fully characterizing the identity of the odorant object given the set of olfactory receptors. The *syntax* of the space of mono-molecular odorants is characterized by the vector of concentration waveforms [u](t) . More details regarding the encoding of mono-molecular odorants by the OSNs is given in Lazar and Yeh (2020) and in the next section below.

### 2.2 Modeling odorant encoding in the antenna

In order to study pure and odorant mixture processing in the Mushroom Body, we first extended our model of the Antenna to account for competitive binding of a mixture of odorant molecules (Nagel and Wilson, 2011; Olsen et al., 2010).

**FIGURE 4:**
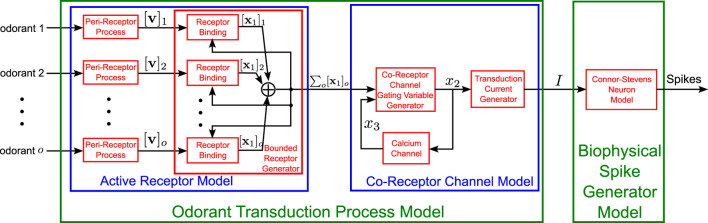
Schematic diagram of the Olfactory Transduction Process for a set O of odorant mixture components, o∈O . The OTP has 3 stages. In the first stage, also known as the active receptor model, each odorant mixture component is processed by a peri-receptor process followed by a feedback controlled receptor binding process that depends on the receptor binding of the other odorant components. The output of the bounded receptor generator is then fed into the second stage, the co-receptor channel model that generates the transduction current. Finally, a biophysical spike generator model converts the transduction current into a spike train. Refer to Table 1 for the mathematical notation.

Odorant molecules are first sensed in either the second-segment of the Antenna or Maxillary-Palp (see Figure 1 2nd column) that are both covered with sensory hairs, called sensilla. Cilia (dendrites) of a few OSNs are housed in each sensillum. Odorants that enter sensilla through the pores on its surface are subsequently transported to the Odorant Receptors (ORs) located on the OSN sensory cilia (Larter et al., 2016). Odorant molecules then bind to the ORs and induce the OSN to generate action potentials. This process is modeled here as the Olfactory Transduction Process (OTP) (see also Figure 4).

We start by briefly reviewing the OTP for a single mono-molecular odorant. The output of the peri-receptor process, that models the overall effect of odorant molecules entering the sensilla before binding to ORs (see also Figure 4), is given by Equation 1 (Lazar and Yeh, 2020):

$$\begin{array}{ll} {\left[v\right]}_{\mathrm{ron}}\left(t\right) & =\text{Re}\left(\int_{R}h\left(t-s\right){\left[u\right]}_{o}\left(s\right)ds\right.\\ & \;+\left.{\left[\gamma \right]}_{\mathrm{ron}}\int_{R}h\left(t-s\right)d{\left[u\right]}_{o}\left(s\right)\right), \end{array}$$ (1)

where ${\left[u\right]}_{o}$ is the concentration of the odorant o , ${\left[v\right]}_{\mathrm{ron}}$ is the concentration profile of the odorant o at receptor r expressed by the OSN n . Re above denotes the rectification function and h(t) is the impulse response of the peri-receptor process.

The bound-receptor model describes the dynamics of binding of odorant molecules to the ORs (see also Figure 4) and is given by

$$\frac{d}{dt}{\left[x_{1}\right]}_{\mathrm{ron}}={\left[b\right]}_{\mathrm{ron}}{\left[v\right]}_{\mathrm{ron}}\left(1-{\left[x_{1}\right]}_{\mathrm{ron}}\right)-{\left[d\right]}_{\mathrm{ron}}\cdot {\left[x_{1}\right]}_{\mathrm{ron}},$$ (2)

where ${\left[x_{1}\right]}_{\mathrm{ron}}$ (bounded between 0 and 1) is the ratio of the ligand-bound receptors bound to the mono-molecular odorant o . The steady-state response is given by Equation 3:

$${\left[x_{1}\right]}_{\mathrm{ron}}=\frac{{\left[a\right]}_{\mathrm{ron}}{\left[v\right]}_{\mathrm{ron}}}{{\left[a\right]}_{\mathrm{ron}}{\left[v\right]}_{\mathrm{ron}}+1},$$ (3)

where ${\left[a\right]}_{\mathrm{ron}}=\frac{{\left[b\right]}_{\mathrm{ron}}}{{\left[d\right]}_{\mathrm{ron}}}$ denotes the odorant affinity. For more details regarding the modeling and biological validation of the mono-molecular OTP, see Lazar and Yeh (2020).

To study odorant mixture representation and processing, we now extended the OTP model to odorant mixtures. In the odorant mixture model, we denote the set of mixture components as O and assume that the odorant components are independent of each other during the peri-receptor process. Receptor r expressed by neuron n can be bound by different odorant components in the mixture, and the ratio of receptors bound by odorant o , denoted as ${\left[x_{1}\right]}_{\mathrm{ron}}$ , is described by

$$\begin{array}{ll} \frac{d}{dt}{\left[x_{1}\right]}_{\mathrm{ron}} & ={\left[b\right]}_{\mathrm{ron}}{\left[v\right]}_{\mathrm{ron}}\left(1-\sum_{p\in O}{\left[x_{1}\right]}_{\mathrm{rpn}}\right)\\ & \;-{\left[d\right]}_{\mathrm{ron}}\cdot {\left[x_{1}\right]}_{\mathrm{ron}},o\in O. \end{array}$$ (4)

Equation 4 models the syntopic interaction between odorants in the mixture and the receptor (Rospars et al., 2008). Note that if only one odorant o is present in the mixture, $\sum_{p\in O}{\left[x_{1}\right]}_{\mathrm{rpn}}$ simply reduces to the single term ${\left[x_{1}\right]}_{\mathrm{ron}}$ as in Equation 2.

It is easy to see that by summing up the Equation 4 over all the odorants present in the mixture, the ratio of the total bound receptors in steady-state amounts to

$$\sum_{o\in O}{\left[x_{1}\right]}_{\mathrm{ron}}=\frac{\sum_{o\in O}{\left[a\right]}_{\mathrm{ron}}{\left[v\right]}_{\mathrm{ron}}}{\sum_{o\in O}{\left[a\right]}_{\mathrm{ron}}{\left[v\right]}_{\mathrm{ron}}+1},$$ (5)

and the steady-state solution to the set of Equations 4 is

$${\left[x_{1}\right]}_{\mathrm{ron}}=\frac{{\left[a\right]}_{\mathrm{ron}}{\left[v\right]}_{\mathrm{ron}}}{\sum_{p\in O}{\left[a\right]}_{\mathrm{rpn}}{\left[v\right]}_{\mathrm{rpn}}+1}.$$ (6)

If we consider the odorant mixture at a particular component ratio as a new “pure” odorant, then we can define, up to a scaling factor, its effective affinity as

$${\left[a\right]}_{rOn}=\frac{\sum_{o\in O}{\left[a\right]}_{\mathrm{ron}}{\left[v\right]}_{\mathrm{ron}}}{\sum_{o\in O}{\left[v\right]}_{\mathrm{ron}}}$$ (7)

The co-receptor channel that models the dynamics of the activation of ligand-gated channels in the mixture model (see also Figure 4) can then be compactly described by

$$\begin{array}{cc} \frac{d}{dt}{\left[x_{2}\right]}_{rOn} & =\alpha_{2}^{O}\left(\sum_{p\in O}{\left[x_{1}\right]}_{\mathrm{rpn}}\right)\left(1-{\left[x_{2}\right]}_{rOn}\right)\\ & \;-\beta_{2}^{O}{\left[x_{2}\right]}_{rOn}-\kappa^{O}{\left[x_{2}\right]}_{rOn}^{2/3}{\left[x_{3}\right]}_{rOn}^{2/3}\\ \frac{d}{dt}{\left[x_{3}\right]}_{rOn} & =\alpha_{3}^{O}{\left[x_{2}\right]}_{rOn}-\beta_{3}^{O}{\left[x_{3}\right]}_{rOn}, \end{array}$$ (8)

where $\alpha_{2}^{O}$ and $\beta_{2}^{O}$ are scalars indicating the rate of activation and deactivation of the gating variable ${\left[x_{2}\right]}_{rOn}$ , respectively, and the constant $\kappa^{O}$ models the calcium feedback for the mixture model. $\alpha_{3}^{O}$ and $\beta_{3}^{O}$ are scalars that indicate the rate of increase and decrease of the gating variable, again for the mixture model. Note that, by using the ratio of the total bound receptors $\sum_{p\in O}{\left[x_{1}\right]}_{\mathrm{rpn}}$ , the receptors bound by different odorants in the mixture jointly determine the dynamics of the gating variable ${\left[x_{2}\right]}_{rOn}$ .

Taken together, the OTP process of an odorant mixture O is given by the following equations

$$\begin{array}{cc} {\left[v\right]}_{\mathrm{ron}}\left(t\right) & =\text{Re}\left(\int_{R}h\left(t-s\right){\left[u\right]}_{o}\left(s\right)ds\right.\\ & \;+\left.{\left[\gamma \right]}_{\mathrm{ron}}\int_{R}h\left(t-s\right)d{\left[u\right]}_{o}\left(s\right)\right),o\in O\\ \frac{d}{dt}{\left[x_{1}\right]}_{\mathrm{ron}} & ={\left[b\right]}_{\mathrm{ron}}{\left[v\right]}_{\mathrm{ron}}\left(1-\sum_{p\in O}{\left[x_{1}\right]}_{\mathrm{rpn}}\right)\\ & \;-{\left[d\right]}_{\mathrm{ron}}\cdot {\left[x_{1}\right]}_{\mathrm{ron}},o\in O\\ \frac{d}{dt}{\left[x_{2}\right]}_{rOn} & =\alpha_{2}^{O}\left(\sum_{p\in O}{\left[x_{1}\right]}_{\mathrm{rpn}}\right)\left(1-{\left[x_{2}\right]}_{rOn}\right)\\ & \;-\beta_{2}^{O}{\left[x_{2}\right]}_{rOn}-\kappa^{O}{\left[x_{2}\right]}_{rOn}^{2/3}{\left[x_{3}\right]}_{rOn}^{2/3}\\ \frac{d}{dt}{\left[x_{3}\right]}_{rOn} & =\alpha_{3}^{O}{\left[x_{2}\right]}_{rOn}-\beta_{3}^{O}{\left[x_{3}\right]}_{rOn}\\ \frac{d}{dt}{\left[I\right]}_{rOn} & ={\left[x_{2}\right]}_{rOn}^{\rho }\left({\bar{I}}_{rOn}-{\left[I\right]}_{rOn}\right)-c^{\rho }{\left[I\right]}_{rOn}. \end{array}$$ (9)

In the last Equation 9, ρ and c are scalars, and ${\bar{I}}_{rOn}$ denotes the maximal amplitude of the current through the co-receptor channel, whose value is empirically determined through parameter sweeping. If the current is activated on a much faster time scale than the activation of the co-receptor, the last equation will operate in steady-state and

$${\left[I\right]}_{rOn}=\frac{{\left[x_{2}\right]}_{rOn}^{\rho }}{{\left[x_{2}\right]}_{rOn}^{\rho }+c^{\rho }}\cdot {\bar{I}}_{rOn}.$$ (10)

Revisiting Equation 9, we note that, similar to the mono-molecular odorant, the encoding of odorant mixtures exhibits multiplicative coupling in a confounding representation of odorant identities and concentration waveforms.

Finally, we note that the spike train generated by the Biophysical Spike Generator (BSG, see also Figure 4) of the OSN expressing receptor r={1,…,R} with noise variance ${\left(\sigma^{O}\right)}^{2}$ in response to the odorant mixture with components in O is given by

$$\underset{k\in Z}{\sum }\delta \left(t-t_{kr}^{O}\right)\leftarrow NoisyConnorStevens\left({\left[I\right]}_{rOn};\sigma^{O}\right),$$ (11)

where ${\left(t_{kr}^{O}\right)}_{k\in Z}$ are the spike times generated by the Noisy Connor-Stevens point-neuron model and δ denotes the Dirac delta functional. Compared with the Connor-Stevens point-neuron (Connor and Stevens, 1971), the Noisy Connor-Stevens point-neuron model exhibits a tunable frequency-current response curve controlled by the variance of the noise. A detailed computational description of the Noisy Connor-Stevens point neuron is available in the Appendix of Lazar and Yeh (2020).

In conclusion, the notation of the key parameters and input/output variables of the Antenna circuit (see Figures 3, 4) are shown in detail in Table 1.

**TABLE 1:** Mathematical notation of the Antenna circuit model.

Symbol	Description
${\left[v\right]}_{o}$	Output of the peri-receptor process (see Equation 1)
${\left[x_{1}\right]}_{o}$	Ratio of receptors bound by odorant o (see Equation 4)
$x_{2}$	Gating variable of the co-receptor channel (see Equation 8)
$x_{3}$	Gating variable of the calcium channel (see Equation 8)
I	Transduction current (see Equation 9)
$\sum_{k}\delta \left(t-t_{k}^{O}\right)$	Spike train response of the OSN (see Equation 11)
r={1,…,R}	Index of receptor types expressed by the OSNs of the Antenna
$\left(b,d,u\left(t\right)\right)$	Tensor modeling the space of odorants presented to the early olfactory system
$\sum_{k\in Z}\delta \left(t-t_{kr}^{O}\right)$	Spike train output of the OSN expressing the r -th receptor type in response to an odorant mixture with components in O
${\left\{\sum_{k\in Z}\delta \left(t-t_{kr}^{O}\right)\right\}}_{r=1}^{R}$	Multi-dimensional spike train output across all OSNs expressing the R different types of olfactory receptors

### 2.3 I/O modeling of the antennal lobe

The Antennal Lobe (AL) can be anatomically divided into some 52 regions called glomeruli, where all the OSNs expressing the same olfactory receptor project their axons into (Buck and Axel, 1991; Firestein, 2001). The dendrites of a uniglomerular projection neurons (uPNs) exclusively innervate a single glomerulus (Scheffer et al., 2020) (see also Figure 1 third column, each color marks the PNs innervating a single glomerulus). Therefore each glomerulus can be considered a separate coding *channel* in which the odorants sensed by a single olfactory receptor type all converge onto the same uPNs. In addition to uPNs, multiglomerular PNs innervate multiple glomeruli and most of them project to the Lateral Horn (LH) while skipping the Mushroom Body (MB). Following (Lazar et al., 2023), multiglomerular PNs are ignored in our AL model described below as physiological recordings are only available for uPNs (Kim et al., 2015). An extensive group of Local Neurons (LNs) exclusively innervates the AL (Scheffer et al., 2020; Lazar et al., 2022). LNs are known to mediate presynaptic inhibition on the OSN axon terminals (Olsen and Wilson, 2008).

**FIGURE 5:**
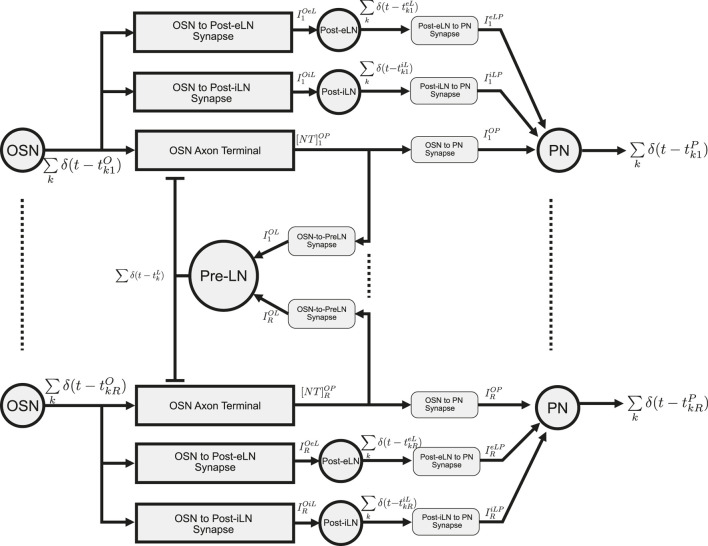
Schematic diagram of the multi-channel AL circuit with spatio-temporal Pre-LN feedback. “OSN” represent a group of OSNs that express the same OR. Their axon terminals provide inputs to uPNs (“PN”) in the same channel (glomerulus). OSN spikes are fed into both Post-eLN and Post-iLN in the channel. These two neurons also drive PNs. In addition to providing inputs to their corresponding PNs, neurotransmitter release at all OSN axon terminals also drive the Pre-LN, which then feedback into the OSN axon terminals. Channels 1 and R are shown. Refer to Table 2 for the mathematical notation. Adapted from Lazar et al. (2023) under Creative Commons Attribution License.

The I/O modeling of the Antennal Lobe is extensively covered in the Supplementary Material, Section 1. Here, we briefly describe the I/O of the Antennal Lobe circuit with spatio-temporal feedback. The schematic diagram of this circuit is shown in Figure 5. This circuit consists of R channels modeling glomeruli (2 channels are shown in Figure 5). As shown in the Supplementary Material, Section 1, each channel r is modeled with 3 Divisive Normalization Processors (DNPs) (Lazar et al., 2023). The first DNP, a model of the OSN Axon Terminal, is controlled by the Presynaptic inhibitory Local Neuron (Pre-LN). The Pre-LN receives inputs from and provides spatio-temporal feedback to all R channels. The OSN Axon Terminal DNP plays a key role in extracting the odorant identity. Each of the other two DNPs models the Postsynaptic excitatory Local Neuron (Post-eLN) and the Postsynaptic inhibitory Local Neuron (Post-iLN), respectively. Their functions are to extract the stimulus onset and offset semantic timing information. Overall, the AL is modeled as a multi-channel DNP with spatio-temporal feedback.

In what follows, in response to the spike train generated by an OSN, we will evaluate the currents injected by each of the three DNPs of a single channel r into a Projection Neuron. For guidance see Figure 5.

The r -th channel parameters of the OSN to Pre-LN synapse are $\left[\alpha_{1}^{OL},\beta_{1}^{OL},{\bar{g}}_{\max}^{OL},E^{OL}\right]$ . The synaptic current $I_{r}^{OL}$ in channel r is described by (see also (the middle of) Figure 5)

$$\frac{d}{dt}x_{r}^{OL}=\alpha_{1}^{OL}\cdot {\left[NT\right]}_{r}^{OP}\cdot \left(1-x_{r}^{OL}\right)-\beta_{1}^{OL}\cdot x_{r}^{OL},$$ (12)

$$I_{r}^{OL}={\bar{g}}_{\max}^{OL}\cdot x_{r}^{OL}\cdot \left(V_{r}^{L}-E^{OL}\right),$$ (13)

where ${\left[NT\right]}_{r}^{OP}$ is the concentration of the synaptic neurotransmitter released by the OSN expressing the r -th receptor and captured by the downstream PN, $V_{r}^{L}$ is the Pre-LN BSG membrane voltage and $E^{OL}$ is the reversal potential of the synapse.

Pre-LN BSG is modeled as a Noisy Connor-Stevens point neuron model (Lazar and Yeh, 2020), similar to the OSN BSGs. The generated spike train is given by

$$\underset{k\in Z}{\sum }\delta \left(t-t_{k}^{L}\right)\leftarrow NoisyConnorStevens\left(\overset{R}{\underset{r=1}{\sum }}I_{r}^{OL};\sigma^{L}\right),$$ (14)

where ${\left(t_{k}^{L}\right)}_{k\in Z}$ are the Pre-LN spike times, and ${\left(\sigma^{L}\right)}^{2}$ is the noise variance of the point neuron model controlling its frequency-current response curve.

The r -th channel parameters of the OSN Axon-Terminal are $\left[\alpha_{1}^{\mathrm{AxT}},\beta_{1}^{\mathrm{AxT}},\kappa_{1}^{\mathrm{AxT}},{\bar{\left[NT\right]}}_{\max}\right]$ , where $\alpha_{1}^{\mathrm{AxT}},\beta_{1}^{\mathrm{AxT}},\kappa_{1}^{\mathrm{AxT}}$ are rate constants and ${\bar{\left[NT\right]}}_{\max}$ denotes the maximum neurotransmitter concentration, and the r -th channel OSN Axon-Terminal is described by

$$\begin{array}{ll} \frac{d}{dt}x_{r}^{\mathrm{AxT}} & =\alpha_{1}^{\mathrm{AxT}}\cdot \underset{k\in Z}{\sum }\delta \left(t-t_{kr}^{O}\right)\cdot \left(1-x_{r}^{\mathrm{AxT}}\right)-\beta_{1}^{\mathrm{AxT}}\cdot x_{r}^{\mathrm{AxT}}\\ & \;-\kappa_{1}^{\mathrm{AxT}}\cdot \underset{k\in Z}{\sum }\delta \left(t-t_{k}^{L}\right)\cdot x_{r}^{\mathrm{AxT}} \end{array}$$ (15)

$${\left[NT\right]}_{r}^{OP}={\bar{\left[NT\right]}}_{\max}\cdot x_{r}^{\mathrm{AxT}}.$$ (16)

where ${\left[NT\right]}_{r}^{OP}$ denotes the vesicle concentration in the OSN Axon-Terminal. Equation 15 describes a temporal feedback Divisive Normalization Processor (DNP) (Lazar et al., 2023) that models the presynaptic normalization taking place at the OSN axon terminal (Olsen and Wilson, 2008). Note that the steady-state response of Equation 15 is of divisive form (see Equation 5 in Supplementary Material; Section 1). The outputs of each OSN Axon-Terminal (feedback DNP) are joined with two additional feedforward DNPs modeled by a Post-eLN and a Post-iLN in each channel (for more details, see Supplementary Material, Section 1). The three DNP outputs in the channel then drive synapses of the Projection Neuron (PN) arborizing the same channel. The total spike train generated by the PN BSG with noise variance ${\left(\sigma^{P}\right)}^{2}$ amounts to

$$\underset{k\in Z}{\sum }\delta \left(t-t_{kr}^{P}\right)\leftarrow NoisyConnorStevens\left(I_{r}^{OP},I_{r}^{\mathrm{eLP}},I_{r}^{\mathrm{iLP}};\sigma^{P}\right),$$ (17)

where $I_{r}^{OP},I_{r}^{\mathrm{eLP}},I_{r}^{\mathrm{iLP}}$ are the synaptic currents from, respectively, the OSN axon terminal, Post-eLN and Post-iLN, and ${\left(t_{kr}^{P}\right)}_{k\in Z}$ are the spike times of the PN (see Figure 5). Details regarding the derivation of the synaptic currents $I_{r}^{OP}$ , $I_{r}^{\mathrm{eLP}}$ and $I_{r}^{\mathrm{iLP}}$ are given in the Supplementary Material, Section 1.

In conclusion, the key parameters and input/output variables of the Antennal Lobe circuit with spatio-temporal feedback (see Figure 5) are shown in detail in Table 2.

**TABLE 2:** Mathematical notation of the Antennal Lobe circuit model.

Symbol	Description
r={1,…,R}	Index of the channels in the spatio-temporal AL circuit
$\sum_{k\in Z}\delta \left(t-t_{kr}^{O}\right)$	Input into the r -th AL channel, where ${\left(t_{kr}^{O}\right)}_{k\in Z}$ are spike times generated by the OSN expressing receptor type r (see Equation 11)
${\left[NT\right]}_{r}^{OP}$	Normalized output signal of the feedback DNP in the r -th channel (see Equation 15, 16)
$\sum_{k\in Z}\delta \left(t-t_{kr}^{P}\right)$	Output of the r -th channel of the AL, where ${\left(t_{kr}^{P}\right)}_{k\in Z}$ are spike times generated by the r -th channel output PN BSG (see Equation 17)
${\left\{\sum_{k\in Z}\delta \left(t-t_{kr}^{P}\right)\right\}}_{r=1}^{R}$	Multi-dimensional output spike trains across all AL channels
$I_{r}^{\mathrm{OeL}}$	Synaptic current to Post-eLN driven by OSN r (see Equations 6–9)
$I_{r}^{\mathrm{OiL}}$	Synaptic current to Post-iLN driven by OSN r (see Equations 11–14)
$\sum_{k}\delta \left(t-t_{kr}^{eL}\right)$	Output of Post-eLN in the r -th channel (see Equation 10)
$\sum_{k}\delta \left(t-t_{kr}^{iL}\right)$	Output of Post-iLN in the r -th channel (see Equation 15)
$I_{r}^{OP}$	Synaptic current into PN driven by OSN axon terminal (see Equations 16, 17)
$I_{r}^{\mathrm{eLP}}$	Synaptic current into PN driven by Post-eLN (see Equations 18, 19)
$I_{r}^{\mathrm{iLP}}$	Synaptic current into PN driven by Post-iLN (see Equations 20, 21)
$\sum \delta \left(t-t_{k}^{L}\right)$	Output of the Pre-LN (see Equation 14)
$I_{r}^{OL}$	Synaptic current to Pre-LN driven by OSN axon terminal in the r -th channel (see Equations 12, 13)
${\left\{\sum_{k\in Z}\delta \left(t-t_{kr}^{O}\right)\right\}}_{r=1}^{R}$	Multi-dimensional input across all AL channels
$x_{r}^{\mathrm{AxT}}$	Normalized output signal of the feedback DNP in the r -th channel
$x^{\mathrm{AxT}}={\left\{x^{\mathrm{AxT}}\right\}}_{r=1}^{R}$	Multi-dimensional normalized output signals of the feedback DNPs across all AL channels

### 2.4 I/O modeling of the mushroom body calyx

The primary circuit architecture of the Mushroom Body Calyx (MB Calyx) exhibits 3 types of neurons. The first neuron type, the uPNs of the Antennal Lobe, projects into the MB Calyx and provides inputs to the second neuron type, the Kenyon Cells (KCs). In the fruit fly, there are about 2,000 KCs on each hemisphere (Li et al., 2020). The connectivity between PNs and KCs is considered random and differs among individual flies (Caron et al., 2013; Masuda-Nakagawa et al., 2005), although a more recent connectome study suggested the existence of more discernible structures (Zheng et al., 2022). Nevertheless, the connectivity is stereotypic with each KC receiving inputs, on average, from 6 to 7 PNs. The third type is an Anterior Paired Lateral (APL) neuron. It covers the entire MB, including the Calyx, and has reciprocal interactions with all the KCs throughout. It has been recently shown that the APL neuron normalizes the magnitude of the overall responses of all the KCs in the MB Calyx (Prisco et al., 2021).

Here, we refine the MB Calyx circuit with two primary structures. First, the PN to KC connectivity is modeled as a bipartite graph, as PNs and KCs can be considered two disjoint sets of vertices in the graph and all edges connect a PN to a KC. Second, we model the interactions between KCs and the APL as a spatio-temporal feedback DNP circuit, similar to the Pre-LN feedback circuit in the Antennal Lobe.

**FIGURE 6:**
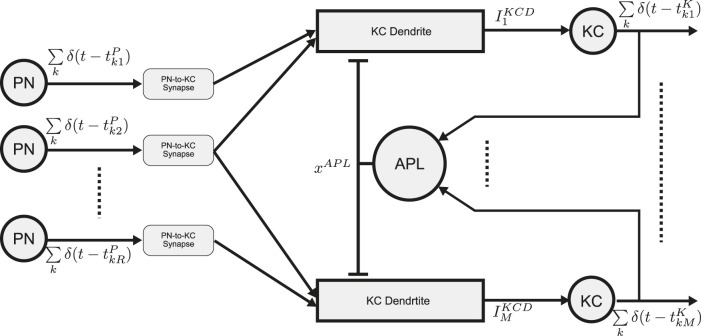
Schematic diagram of the Calyx circuit with spatio-temporal APL feedback. Spiking outputs of the PNs provide inputs to KC dendrites. Each KC receives inputs from Q PNs. Output of the KC dendrites then drive their respective KC BSGs (“KC”) to respond and their spikes are fed into the APL neuron. The APL neuron output provides a second input to each KC dendrite. Refer to Table 3 for the mathematical notation.

The schematic diagram of the MB Calyx circuit with spatio-temporal Anterior Paired Lateral (APL) feedback is shown in Figure 6. The m -th KC dendritic output current $I_{m}^{\mathrm{KCD}}$ (superscript “KCD” for “KC Dendrite”) is determined by the input of a random number of PN axons projecting into each KC dendritic tree and the feedback $x^{\mathrm{APL}}$ provided by the APL neuron.

The output current $I_{m}^{\mathrm{KCD}}$ of the *m*th KC dendrite with parameters $\left[\alpha_{1}^{\mathrm{KCD}},\beta_{1}^{\mathrm{KCD}},\kappa_{1}^{\mathrm{KCD}}\right]$ is described by

$$\begin{array}{ll} \frac{d}{dt}x_{m}^{\mathrm{KCD}} & =\alpha_{1}^{\mathrm{KCD}}\cdot \underset{r\in I_{m}}{\sum }\underset{k\in Z}{\sum }\delta \left(t-t_{kr}^{P}\right)\cdot \left(1-x_{m}^{\mathrm{KCD}}\right)\\ & \;-\beta_{1}^{\mathrm{KCD}}\cdot x_{m}^{\mathrm{KCD}}-\kappa_{1}^{\mathrm{KCD}}\cdot x_{m}^{\mathrm{KCD}}\cdot x^{\mathrm{APL}} \end{array}$$ (18)

$$I_{m}^{\mathrm{KCD}}={\bar{I}}_{\max}^{K}\cdot x_{m}^{\mathrm{KCD}},$$ (19)

where $I_{m}$ denotes a set of PNs connected to the dendrite of the *m*th KC, $\alpha_{1}^{\mathrm{KCD}},\beta_{1}^{\mathrm{KCD}},\kappa_{1}^{\mathrm{KCD}}$ are rate constants. Here, we assume $I_{m}$ to be a random set (Caron et al., 2013). The number of PN inputs that a KC receives is parameterized by Q .

For simplicity, the APL feedback signal $x^{\mathrm{APL}}$ is modeled as the solution of a kinetic equation with parameters $\left[\alpha_{1}^{\mathrm{APL}},\beta_{1}^{\mathrm{APL}}\right]$ driven by the aggregated input KC spike trains $\sum_{m=1}^{M}\sum_{k\in Z}\delta \left(t-t_{km}^{K}\right)$ :

$$\begin{array}{ll} \frac{d}{dt}x^{\mathrm{APL}} & =\alpha_{1}^{\mathrm{APL}}\cdot \overset{M}{\underset{m=1}{\sum }}\underset{k\in Z}{\sum }\delta \left(t-t_{km}^{K}\right)\cdot \left(1-x^{\mathrm{APL}}\right)\\ & \;-\beta_{1}^{\mathrm{APL}}\cdot x^{\mathrm{APL}}. \end{array}$$ (20)

where $\sum_{m=1}^{M}\sum_{k\in Z}\delta \left(t-t_{km}^{K}\right)$ is the total KC spiking activity. For simplicity, we omit the PN-KC and KC-APL synaptic dynamics.

The KC BSG is modeled by the NoisyConnorStevens point neuron, with noise variance ${\left(\sigma^{K}\right)}^{2}=0$ , and generated spike train

$$\underset{k\in Z}{\sum }\delta \left(t-t_{km}^{K}\right)\leftarrow NoisyConnorStevens\left(I_{m}^{\mathrm{KCD}};\sigma^{K}\right),$$ (21)

where ${\left(t_{km}^{K}\right)}_{k\in Z}$ are the spike times generated by the *m*th KC neuron and δ denotes the Dirac functional.

In conclusion, the key parameters and input/output variables of the Calyx circuit (see Figure 6) are shown in detail in Table 3.

**TABLE 3:** Mathematical notation of the Mushroom Body Calyx circuit model.

Symbol	Description
m={1,…,M}	Index of the KC neurons in the Calyx circuit
r={1,…,R}	Index of the PNs
$\sum_{r\in I_{m}}\sum_{k\in Z}\delta \left(t-t_{kr}^{P}\right)$	Input to the m -th KC dendrite, where $I_{m}$ denotes a random set of PNs connected to the m -th KC dendrite and ${\left(t_{kr}^{P}\right)}_{k\in Z}$ is the set of spike times generated by the PN BSG at the output of the r -th AL channel
${\left\{\sum_{r\in I_{m}}\sum_{k\in Z}\delta \left(t-t_{kr}^{P}\right)\right\}}_{r=1}^{R}$	Multi-dimensional input to the dendrites across all KC neurons
$x_{m}^{\mathrm{KCD}}$	Normalized dendritic output current of the m -th KC neuron
$x^{\mathrm{KCD}}={\left\{x_{m}^{\mathrm{KCD}}\right\}}_{m=1}^{M}$	Multi-dimensional normalized dendritic output current across all KC neurons
$\sum_{k\in Z}\delta \left(t-t_{km}^{K}\right)$	Output of the m -th KC neuron, where ${\left(t_{km}^{K}\right)}_{k\in Z}$ is the set of spike times generated by the m -th KC BSG
${\left\{\sum_{k\in Z}\delta \left(t-t_{km}^{K}\right)\right\}}_{m=1}^{M}$	Multi-dimensional output across all KC neurons
$\sum_{k}\delta \left(t-t_{kr}^{P}\right)$	PN spike outputs
$I^{\mathrm{KCD}}$	Synaptic outputs of the KC dendrite (see Equation 18, 19)
$\sum_{k}\delta \left(t-t_{km}^{K}\right)$	KC spike output

## 3 I/O characterization of odor signal processing in the MB calyx

In what follows, our goal is to characterize the I/O of the MB Calyx, the last building block of the OEM cascade depicted in Figure 2. Given the prior modeling of the space of odorants in Section (2.1), the odorant encoding process in the Antenna described in Section (2.2) and, the odor signal processing taking place in the Antennal Lobe and detailed in Section (2.3), the input to the Mushroom Body Calyx can be readily evaluated as the PN response at the output of the Antennal Lobe for pure and odorant mixtures.

Recall that, we evaluated the odorant encoding process described in Section (2.2) with 110/23 odorant/receptor pairs stored in the DoOR dataset (Münch and Galizia, 2016). Each of the 110 odorants was associated with a 23-dimensional affinity vector whose entries were estimated using the algorithm advanced in (Lazar and Yeh, 2020). Given the PN output provided by the Antennal Lobe model (Lazar et al., 2023), we shall investigate whether the Mushroom Body Calyx extracts semantic information, i.e., the identity of pure odorants and odorant mixtures, faithfully and distortion free.

This section is organized as follows. In section 3.1 we evaluate the effect of the PN-KC connectivity on the KC dendritic input for both pure (mono-molecular) and odorant mixtures. In section 3.2 we evaluate the effect of the KC-APL feedback on the KC dendritic output for both pure and odorant mixtures. Finally, in section 3.3 we show how the Calyx extracts and represents semantic information in the spike domain.

### 3.1 The effect of the PN-KC connectivity on the KC dendritic input for pure and odorant mixtures

A key descriptor of the Calyx circuit is the connectivity between PNs and KCs, i.e., the adjacency matrix of the PN-KC bipartite graph. The topology of the bipartite graph is determined by two factors. First, each KC receives inputs from a number of Q PNs. Second, the PNs are randomly selected in an individual fly (Caron et al., 2013). This determines how the KC dendritic trees sample the R -dimensional space of the PN responses to odorants.

We first evaluate the dependency of the KC dendritic inputs on Q . Biologically, the value of Q corresponds to the number of claw-like endings of the KC dendrites (Schürmann, 1974; Yusuyama et al., 2002). Each KC claw receives dense synaptic inputs mostly from a single PN. Therefore, the number of dendritic claws of a KC largely determines the number of different PNs that the KC receives inputs from.

A recent experimental study has examined the effect of the number of KC claws on fly’s ability to discriminate odorants (Ahmed et al., 2023). Genetic manipulation allowed the authors to obtain flies that have an increased or decreased number of dendritic claws. Here we evaluate the effect of computationally changing the value of Q .

#### 3.1.1 The effect of the PN-KC connectivity on the dendritic KC input for pure odorants

**FIGURE 7:**
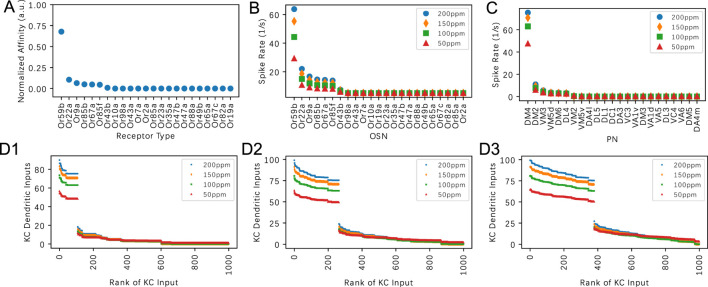
Evaluating the effect of the PN-KC connectivity parameter Q on the dendritic KC input for Acetone. **(A)** Acetone affinity in descending (ranking) order. Affinity is normalized by the sum of all affinity values across receptor types. **(B)** Steady-state responses of OSNs to Acetone at four different constant amplitude concentration levels. **(C)** Steady-state responses of PNs to Acetone at four different constant concentration levels. **(D)** Dendritic inputs to each KC in descending order of input strength, at four different different constant concentration levels. **(D1)** Q = 3, **(D2)** Q = 6, **(D3)** Q = 9. The horizontal axis lists the KCs in ranking order.

In this section we evaluate the dependence of the KC dendritic inputs on Q (number of claw-like endings of the KC dendrites) for pure odorants. In Figure 7, we evaluate our model for Acetone at four constant amplitude concentration levels: 50ppm, 100ppm, 150ppm and 200ppm, and examined the respective steady-state responses at the OSNs, PNs and KC dendritic inputs. In Figure 7A, the affinity value of each of the 23 receptors normalized by the sum of all affinity values is shown in descending (ranking) order. Note that the responses presented in ranking order provide a more intuitive representation of the structure of the response vectors. The OSN and PN spike train responses are shown in Figures 7B, C, respectively. Consistent with (Lazar et al., 2023), while both OSN and PN responses are sensitive to odorant concentration, the dependency at the PN level is markedly reduced.

Visualizing the KC responses in Figures 7D1–D3, we observe that the number of KCs activated by a given odorant is strongly influenced by the Q values: Q=3 , Q=6 and Q=9 .

We note that the ranking of the KC dendritic inputs is largely determined by the number of top responding PNs. For example, if the Q=6 inputs to a KC originate from the top 6 responding PNs, then that KC is ranked tops among all other KCs. Since only 1 PN (DM4 PN) out of the 23 PNs strongly responds to Acetone, the KCs that receive inputs from the DM4 PN have significantly higher total dendritic input than the other KCs (see also Figures 7D1–D3). This results in a large gap in the dendritic input-rank curve. As Q increases from 3 to 9, the number of KCs that have DM4 PN dendritic input also increases. This increase leads to a larger percentage of KCs with larger inputs while the total number of KCs remains unchanged.

**FIGURE 8:**
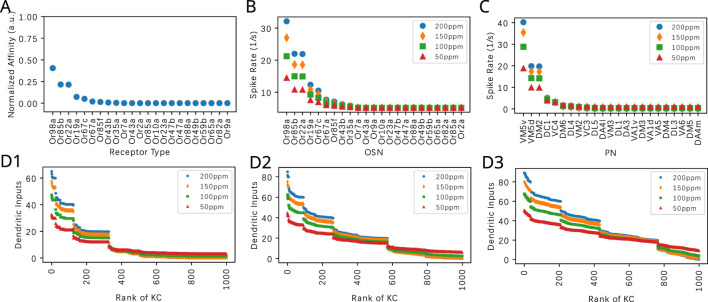
Evaluating the effect of the PN-KC connectivity parameter Q on the dendritic KC input for Nerol. **(A)** Nerol affinity in descending order. Affinity is normalized by the sum of all affinity values across receptor types. **(B)** Steady-state responses of OSNs to Nerol at four different constant amplitude concentration levels. **(C)** Steady-state responses of PNs to Nerol at four different constant concentration levels. **(D)** Dendritic inputs to each KC in descending order of input strength, at four different constant concentration levels. **(D1)** Q = 3, **(D2)** Q = 6, **(D3)** Q = 9. The horizontal axis lists the KCs in ranking order.

In Figure 8, we characterize responses to the odorant Nerol in the same way as in Figure 7 for the odorant Acetone. We note that the affinity values of 3 receptors are relatively higher. This creates a different signature in the ordered ranking of the KC inputs. The general trend is similar to the case when Acetone is presented. With a smaller Q value, less KCs receive enough inputs to generate spikes, as experimentally observed in Ahmed et al. (2023).

With an increasing number of PNs responding to a pure odorant, the dendritic input-ranking curve becomes smoother. See, for example, the results for Diethyl Succinate and Ethyl Butyrate shown, respectively, in Supplementary Figures S3, S4. Ethyl Butyrate elicits responses in a wide range of PNs, and the dendritic input-rank curves are smoother without noticeable gaps between KC dendritic inputs.

Similar dendritic input-rank plots can be obtained for *randomly* instantiated PN-KC bipartite graphs (see Supplementary Figure S5). Note, however, that for random connectivity, the exact ranking order of each KC might differ. Since the connectivity between PNs and KCs has been shown to be random and may differ from fly to fly (Caron et al., 2013), the preservation of the input-ranking for different odorants across concentration amplitudes applies across individual flies.

#### 3.1.2 The effect of the PN-KC connectivity on the KC dendritic input for odorant mixtures

**FIGURE 9:**
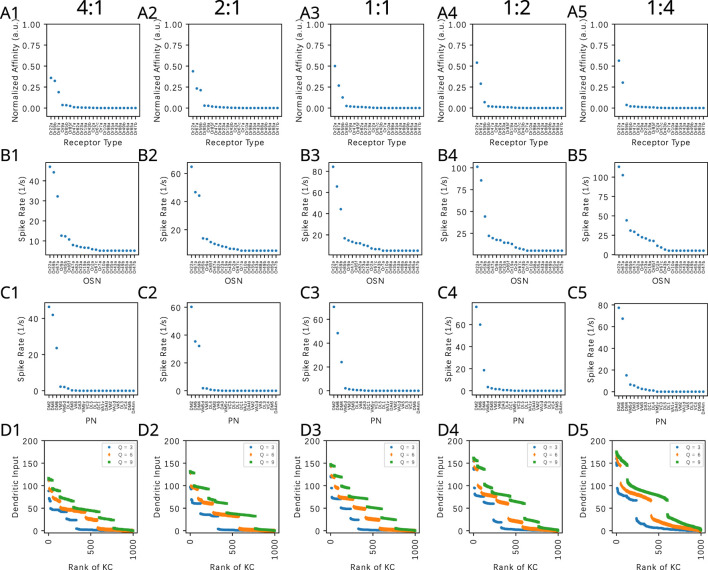
Evaluating the effect of the PN-KC connectivity parameter Q on the dendritic KC input with mixtures of Acetone and Diethyl Succinate. Concentration of Acetone is kept at 100ppm, and the concentration of Diethyl Succinate changes in each column such that the ratio of the two odorants are (column 1) 4:1, (column 2) 2:1, (column 3) 1:1, (column 4) 1:2 (column 5) 1:4. The mixtures are presented at constant concentration levels, and the steady-state responses are shown. **(A)** Effective affinity of the mixture at different component ratios. **(B)** OSN responses. **(C)** PN responses. **(D)** KC dendritic inputs for (blue) Q=3 , (orange) Q=6 and (green) Q=9 . The horizontal axis lists the KCs in ranking order.

In Figure 9, we evaluate the dependence of the KC dendritic inputs on the connectivity parameter Q when a binary odorant mixture consisting of Acetone and Diethyl Succinate is presented. The concentration of Acetone is kept at 100ppm, and the concentration of Diethyl Succinate changes in each column such that the ratio of the two odorants are, respectively, 4:1, 2:1, 1:1, 1:2 and 1:4. The mixtures are presented at constant amplitude concentration levels, and the steady-state responses are shown.

We observe that across all Q values, the ordered KC dendritic input exhibits different characteristics when the component concentration amplitude ratio shifts from 4:1 to 1:4. This characteristic is largely preserved for different Q values at a particular component concentration ratio. In particular, the range of magnitude of KC dendritic inputs are similar across 3 Q values, as there are only 3 large components in the affinity vectors.

Concluding, the connectivity between the PNs and KCs modeled by a bipartite graph with parameter Q changes the distribution of the ranking of the output of dendritic KCs. In Figures 7–9 higher rank KC input values gravitate and are grouped together. These groupings can be more easily distinguished from lower rank values that also gravitate together. In addition, these response properties are preserved despite the randomness of the connectivity between PN and KC across individual flies.

### 3.2 The effect of the KC-APL feedback on the KC dendritic output for pure and odorant mixtures

In this section we analyze the dependence of the Mushroom Body Calyx circuit on the APL feedback. We focus on the effect of APL feedback on the KC dendritic outputs that drive the KC spike generation. For simplicity, we set the connectivity parameter of the PN-KC bipartite graph to Q=6 , a number consistent with average of PN-to-KC connections observed in the connectome (Scheffer et al., 2020). We show that the APL feedback facilitates the extraction of semantic odorant information by normalizing KC responses and by reducing odorant concentration dependence of the KC dendritic output.

#### 3.2.1 The effect of the KC-APL feedback on the KC dendritic output for pure odorants

We first note that the differential DNP described by Equations 18, 20 is, in steady-state, approximately characterized by a monotonically increasing sigmoid function of KC dendritic inputs. Therefore, we expect that the ranking of the magnitude of KC dendritic inputs is preserved by the KC dendritic outputs.

**FIGURE 10:**
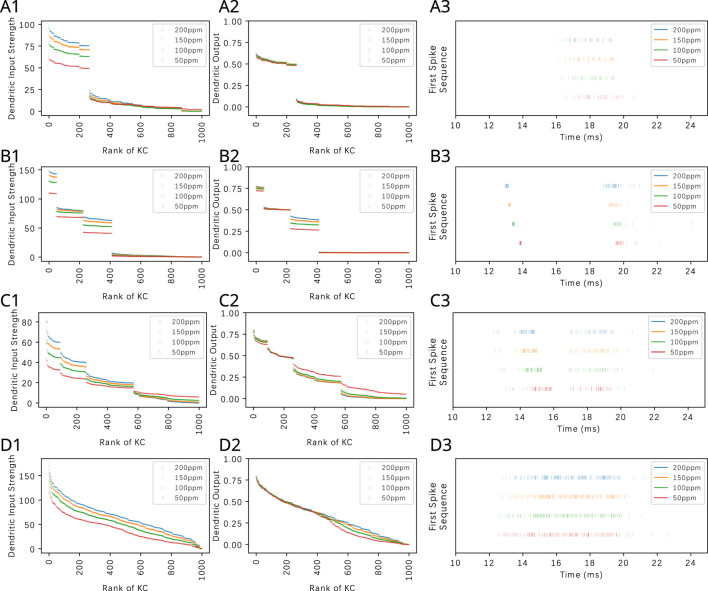
APL feedback facilitates the extraction of odorant semantic information by normalizing KC responses and by reducing odorant concentration dependence of the KC dendritic output. The connectivity parameter of the PN-KC bipartite graph is Q=6 . Odorant semantics in row **(A)** Acetone, **(B)** Diethyl Succinate, **(C)** Nerol, and **(D)** Ethyl Butyrate. (left column) Ranking of KC dendritic inputs. (middle column) Ranking of KC dendritic outputs. (right column) Odorant semantics encoded in the time domain across the population of KCs. The first spikes of each of the active KCs in response to each odorant are collected onto a single row for each of the odorant concentration amplitude values.

In Figure 10, we depict the transformation of KC dendritic inputs (left column) into dendritic outputs (middle column) in the presence of APL feedback. Each row of Figure 10 shows the transformation for one of the four odorants that we tested (Acetone, Diethyl Succinate, Nerol and Ethyl Butyrate) each with four different constant amplitude concentration values. The dendritic output amounts to $x_{m}^{\mathrm{KCD}}$ in steady-state. Here the KC spiking threshold was chosen to be 0.5. Thus, the KCs that have dendritic output greater than 0.5 will generate spikes that contribute to the magnitude of the amplitude of the APL feedback.

As shown in Figure 10, the presence of APL feedback largely removes the concentration dependence of the KC dendritic output if the latter is above threshold. This demonstrates that the proposed divisive normalization circuit is capable of further reducing the variability of KC responses to odorants of different concentration levels (Prisco et al., 2021) beyond the normalization effect induced by the Local Neurons of the Antennal Lobe (Lazar et al., 2023), thereby further separating odorant semantic information from syntactic information (Lazar et al., 2023). The aggregation of the KC responses in Figure 10 (right column) will be discussed in Section 3.3.

#### 3.2.2 The effect of the KC-APL feedback on the KC dendritic output for odorant mixtures

**FIGURE 11:**
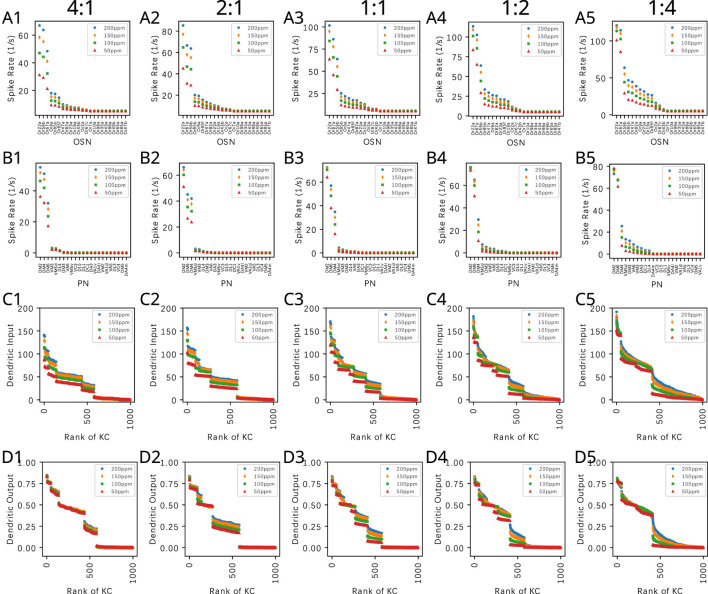
By normalizing KC responses and by reducing odorant concentration dependence of the KC dendritic output, APL feedback reduces the variability of KC responses to component concentration ratios of odorant mixtures. **(A)** OSN responses to mixture of Acetone and Diethyl Succinate at five different component ratios and different total concentration levels. Legend shows the concentration of Acetone. Concentration of Diethyl Succinate can be derived from the component ratio. **(B)** PN responses. **(C)** KC dendritic inputs. **(D)** KC dendritic outputs. Ratios of acetone to Diethyl Succinate are (column 1) 4:1, (column 2) 2:1, (column 3) 1:1, (column 4) 1:2, (column 5) 1:4. The horizontal axes in rows **(C)** and **(D)** list the KCs in ranking order. The connectivity parameter of the PN-KC bipartite graph is Q=6 .

APL feedback is equally effective for extracting the semantic information of odorant mixtures. In Figure 11, we consider a binary mixture consisting of Acetone and diethyl succinate at different component constant amplitude concentration ratios. For each component ratio, we also varied the total concentration while keeping the ratio fixed. The OSN responses to the mixtures are shown in Figure 11A. PN responses, as shown in Figure 11B, exhibited reduced variability to constant concentration ratios. The KC dendritic inputs and and the dendritic outputs are, respectively, shown in Figures 11C. While the magnitude of dendritic inputs varies across component ratios and total concentration, the dendritic outputs display a markedly reduced variability across concentration amplitudes. Among the different component ratios tested, the overall range of responses at the KC dendritic outputs are also similar.

Normalized KC dendritic outputs naturally maintain the number of active KCs with a single spiking threshold. From Figures 10, 11, we can see that about 20% of the KCs are above the spiking threshold. With a different threshold or Q value, the percentage of active KCs can easily be controlled. This demonstrates that the spatio-temporal DNP model of the MB Calyx circuit is a more natural mechanism for ensuring the sparsity of KC responses, as opposed to an artificial winner-take-all mechanism that has been used by other models of the mushroom body for enforcing the sparseness of KC responses (Dasgupta et al., 2017; Saumweber et al., 2018; Gkanias et al., 2022).

### 3.3 The Calyx extracts and represents odorant semantic information in the spike domain

Ranking the dendritic input and output KCs in Figures 10, 11 provides insights into the structure of the affinity vector of pure and mixture odorants under consideration. The ranking operation, however, exhibits a combinatorial complexity. This forbidding complexity can be computationally readily avoided by mapping, for each KC, the dendritic output into the spike domain. The proposed code takes the first spike of each active KC and joins them all together at generation time into a single first spike sequence. Figure 10 (right column) shows the first spike sequences for four different odorants, each at four different concentration levels. We note that these spike sequences are not generated by a single neuron. Rather, each sequence consists of a train of spikes received by, e.g., a Mushroom Body Output Neuron (MBON) (or APL neuron) innervating its presynaptic KCs in a MB compartment. Therefore, the order of the KC dendritic output that is invariant to odorant concentration can be naturally read out by an MBON (or APL) based on the timing of the proposed first spike sequence.

Since the KC dendritic output is largely concentration invariant for the KCs with dendritic output above the threshold, the variability of the sequence of spikes across a range of concentration amplitude values is small. The first spike sequences in Figure 10 (right column) are clearly different when due to two different odorants but rather similar when due to two different concentration waveforms of the same odorant. In the Supplementary Figure S6, we display the ranked KC dendritic inputs, the ranked KC dendritic outputs, the first spike sequence and the cumulative interspike intervals for all 110 odorants whose OSN responses have been characterized for 23 ORs at a single concentration level in the DoOR dataset (Münch and Galizia, 2016). Note that the cumulative interspike distance plots are largely concentration invariant. This is amply displayed in the last column of the Supplementary Figure S6 for 110 mono-molecular odorants evaluated at four different concentration amplitude values. Thus, we hypothesize that the sequence of first spikes generated by each individual KC represents the odorant semantic information in the time domain largely unaltered by the syntactic information of the odorant concentration waveform.

The key advantage of the first spike sequence code across the active KCs in the spike domain is that the readout of the sequence of spikes arriving at the MBONs does not require the knowledge of the KCs that the spike originated from. The entire sequence becomes a single code. Therefore, the code remains the same for different flies with different instantiations of the PN-KC bipartite graph.

**FIGURE 12:**
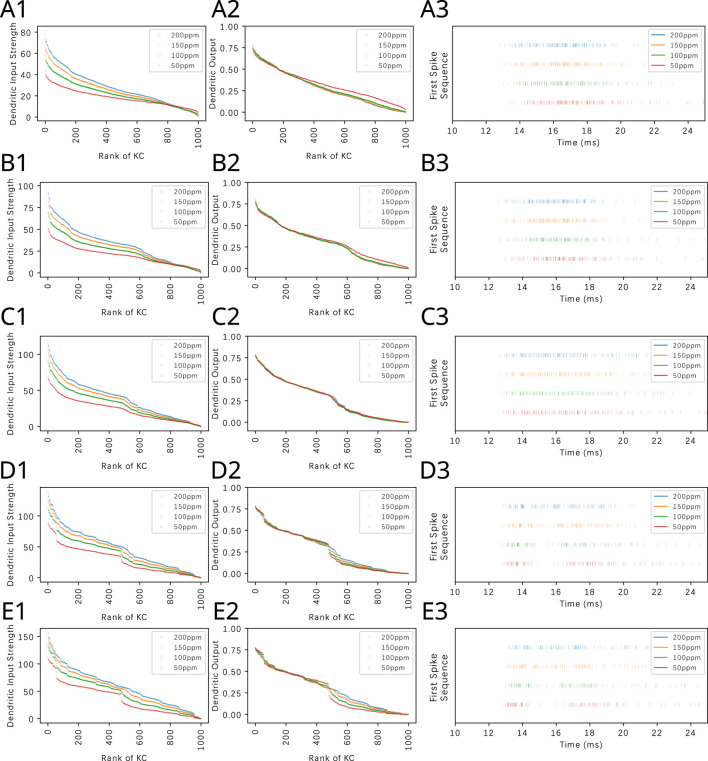
Odorant semantics information of a mixture of Methanol and Benzyl Alcohol with different constant concentration amplitude ratios encoded in the time domain across the population of KCs. The mixtures are presented at a fixed ratio in row **(A)** 4:1, **(B)** 2:1, **(C)** 1:1, **(D)** 1:2 and (E) 1:4. For each fixed ratio, 4 Methanol concentration levels are used, (red) 50ppm, (green) 100ppm, (orange) 150ppm and (blue) 200ppm. (left column) Ranking of KC dendritic inputs. (middle column) Ranking of KC dendritic outputs. (right column) Odorant semantics encoded in the time domain across the population of KCs. The first spikes of each of the active KCs in response to each odorant are collected onto a single row for each of the odorant concentration amplitude values.

The first spike sequence code can also be used to distinguish odorant mixtures with different mixture ratios. Figure 12 (right column) shows the first spike sequence code for mixtures of Methanol and Benzyl Alcohol at five different ratios. For each fixed ratio, the concentration of the mixture components are presented at four different Methanol concentration levels. Again, the first spike sequence code shows different patterns for each ratio but similar patterns for different concentration ampitudes of the same ratio. The corresponding cumulative interspike intervals are shown in Supplementary Figure S7. The response of the OEM to two other binary mixtures are shown in Supplementary Figures S8, S9.

## 4 Discussion

The early olfactory sensory circuits evolved to encode and identify odorants in various ecological niches, thereby raising the structure and features of the odorant space to be key determinants of the encoding mechanisms adapted in the olfactory pathways. Despite its importance, however, an explicit modeling of the odorant space has often been neglected when discussing odor signal processing in the early olfactory circuits (Endo and Kazama, 2022).

In the present work, we explicitly modeled odorant stimuli in terms of their semantic and syntactic information content, and explored how the early olfactory system of the fruit fly separates semantic and syntactic information. Recall that, Shannon (Shannon, 1948) made a clear distinction between meaning (semantic) and syntactic information. He felt, rightly so, that syntactic information can be formalized and that led to, among others, key concepts in information theory such as channel capacity, coding theorems, etc. One of his main arguments was that “a bit is a bit” and there is no meaning associated with “bits”. He did not address the challenges of formalizing the concept of semantic information.

In accordance to Shannon’s distinction between syntax and semantics, our key prior research results (Lazar and Yeh, 2020; Lazar et al., 2023) have pointed out that the traditional application of methods of information theory, signal processing and control theory to odor signal processing is lacking the notion of “meaning” or semantics. An example might help clarify our point of view. When a neuroscientist applies a mono-molecular odorant such as Acetone, to the Antennae of the fruit fly, and only provides single OSN recordings to a second neuroscientist without mentioning the odorant identity, the recordings alone provide no clues that the odorant in question is Acetone. This is because different odorant identity and concentration pairs can lead to the same OSN spike train response (Lazar and Slutskiy, 2012). Most of the experiments in the olfactory literature, assume that the odorant identity is known. As such, prior arts (Blazing and Franks, 2020; Endo and Kazama, 2022; Bandyopadhyay and Sachse, 2023) have primarily focused on the representation of odorant syntactic information (i.e., concentration amplitude) and cannot, therefore, serve as baseline methods without a formal computational/theoretic model of odorant identity. In contrast we argue that odor signal processing in the Early Olfactory System (EOS) of the fruit fly, is mostly focussed on extracting semantic information. Consequently, we argue that olfactory research needs to shift from solely focusing on processing syntactic (or Shannon) information to processing semantic, i.e., odorant identity information.

To that end, by extending our previous work on the functional logic of odor signal processing in the Antennal Lobe (Lazar et al., 2023), we have established that the Antennal Lobe and Calyx jointly remove the concentration dependency of the odorant information from the confounding representation of the Antenna (Lazar and Yeh, 2020). We demonstrated that these circuits separate the odorant semantics from syntax, thereby undoing the multiplicative coupling of these two information streams in the Antenna.

We showed that in the Calyx the sought after semantic information underlies the ranking of the KC dendritic output after the KC dendritic input undergoes the PN-KC random connectivity and the spatio-temporal feedback provided by the APL neuron. Consequently, expansion recoding in the Calyx characterizes the structure of vector PN responses by computing fixed mean random additive combinations, and use their ranking as a simple yet powerful way of extracting the semantic information of the odorant. More importantly, we addressed the combinatorial complexity of ranking by mapping, for each KC, the concentration-invariant dendritic output into the spike domain. The proposed time code takes the first spike of each active KC and joins them all together at generation time into a single first spike sequence. Clearly, the order of the first spikes across the population of KCs reflects the ranking order at negligible complexity. The existence of such concentration-invariant spike code is supported by increasing evidence in the Antenna and Antennal Lobe (Haddad et al., 2013; Wilson et al., 2017; Zwicker, 2019; Chong et al., 2020; Egea-Weiss et al., 2018), the piriform cortex (Bolding and Franks, 2018), the visual system (Rullen and Thorpe, 2001; Gollisch and Meister, 2008), and at the neuron level in general (Branco et al., 2010).

*Time* is an intrinsic variable of the concentration waveform, but not of the odorant object identity. Interestingly, the key result of the modeling and characterization of the early olfactory system we advanced here asserts that the semantic information is mapped into the time domain by the Calyx circuit, in the form of the first spike sequence code. This allows a low complexity single readout of the semantic information at the downstream MBONs regardless of the exact connectivity between PNs and KCs in individual flies. The code itself is temporally bounded, making it possible for timely memory access in the MB compartments.

Overall, our work argues that the main information pattern processed by the early olfactory system is supplied by the odorant semantics and not the syntax. The odorant semantics is mapped by the Calyx circuit into a first spike sequence in the time domain. This is, clearly, central to understanding the functional logic of the neural circuits involved in odor signal processing in the EOS of the fruit fly brain. Our approach, backed up by the analysis of the fist spike sequence code and the robustness of the cumulative interspike intervals of 110 odorants in the DoOR dataset (Münch and Galizia, 2016), represents a radical departure in understanding the logic of odor signal processing in the EOS. Among others, it calls for recordings of the KCs in the MB with the ultimate goal of addressing the existence of the first spike sequence code that we advanced here.

Furthermore, we extended the model of mono-molecular odor signal processing in the Antenna, Antennal Lobe and Calyx to odorant mixtures. Our model covers the syntopic interactions (Rospars et al., 2008) among odorants competing for the unbound receptors in the OSN cilia while abstracting additional resources (e.g., the number of permeable pores on the surface of sensilla binding proteins (Larter et al., 2016)) into the peri-receptor processes. No further interactions between odorants and the same receptor type have been modeled that may result in binding/dissociation facilitation or suppression (Singh et al., 2019). We note that alternative extensions to the OTP model may be developed for describing other phenomena of odorant mixture encoding, such as *masking* (Reddy et al., 2018), and *ephaptic coupling* (Su et al., 2012; Wu et al., 2022; Pannunzi and Nowotny, 2021). However, despite recent insight into the structure of *Machili hrabei* olfactory receptor (del Mármol et al., 2021), additional recording datasets are required to determine which model most accurately describes odorant mixture binding to *Drosophila* olfactory receptors.

Algorithmically, our model of the first three stages of the EOS anchors on Divisive Normalization Processors (DNPs). DNPs are models of biological neural circuits (Lazar et al., 2020b; Lazar and Zhou, 2023) with spatio-temporal feedforward and/or feedback control. The power of DNPs in modeling key computational building blocks in the early olfactory system suggests their applicability in many other sensory processing systems in and beyond those of the fruit fly brain.

## Data Availability

The original contributions presented in the study are included in the article/Supplementary Material, further inquiries can be directed to the corresponding author.
