# Supplementary material for: Modeling and characterization of pure and odorant mixture processing in the Drosophila mushroom body calyx
Source: Front Physiol. 2024 Oct 16;15:1410946. doi: 10.3389/fphys.2024.1410946 (PMC11521939; doi:10.3389/fphys.2024.1410946)
Supplement: Supplementary file 1 [file DataSheet1.pdf]

frontiers

Supplementary Material

1 I/O Modeling of the Antennal Lobe

In this section we present a detailed I/O modeling of the Antennal Lobe circuit. We start by modeling the I/O of the single-channel temporal (see Section 1.1) feedback configuration followed by the multi-channel spatio-temporal (see Section 1.2) feedback configurations. More details can be found in (Lazar et al., 2023).

1.1 I/O Modeling of a Single-Channel Antennal Lobe with Temporal Feedback

The channel considered here is generic and will not be indexed. This reduces the burden in notation. In section 1.2 the channels will be indexed by the receptor index $r = \{1, \ldots, R\}$. The single channel AL circuit with temporal feedback consists of 3 subcircuits with feedback Pre-LN, with feedforward Post-eLN and with feedforward Post-iLN. Note that Pre-LN denotes a presynaptic Local Neuron that acts presynaptically on the OSN axon terminals. The Post-eLN and Post-iLN denote, respectively, an excitatory Local Neuron and an inhibitory Local Neuron in the Antennal Lobe that act postsynaptically on PNs.

Single-Channel Antennal Lobe Subcircuit with Feedback Pre-LN

The OSN to Pre-LN synapse in Figure S1 (top left) with parameters $[\alpha_1^{OL}, \beta_1^{OL}, \bar{g}_{max}^{OL}, E^{OL}]$ is described by

$$\frac{d}{dt}x^{OL} = \alpha_1^{OL} \cdot [NT]^{OP} \cdot (1 - x^{OL}) - \beta_1^{OL} \cdot x^{OL} \tag{1}$$

$$I^{OL} = \bar{g}_{max}^{OL} \cdot x^{OL} \cdot (V^L - E^{OL}), \tag{2}$$

where $[NT]^{OP} = \overline{[NT]}_{max} \cdot x^{AxT}$ is the concentration of the synaptic neurotransmitter, I^{OL} is the synaptic current injected into the Pre-LN BSG, V^L is the Pre-LN membrane voltage, and E^{OL} is the reversal potential of the synapse. The normalized neurotransmitter concentration x^{AxT} is described by eq.(3) below. For the Pre-LN BSG model the noise variance is $(\sigma^{OL})^2 = 0$, and the spike train generated by the Pre-LN amounts to

$$\sum_{k\in\mathbb{Z}} \delta(t - t_k^L) \leftarrow \text{NoisyConnorStevens}(I^{OL}; \sigma^{OL}).$$

The output $[NT]^{OP}$ of the (feedback) OSN Axon-Terminal model with parameters $[\alpha_1^{AxT}, \beta_1^{AxT}, \kappa_1^{AxT}, \overline{[NT]}_{max}]$ is of the form $[NT]^{OP} = \overline{[NT]}_{max} \cdot x^{AxT}$ where

$$\frac{d}{dt}x^{AxT} = \alpha^{AxT} \cdot \sum_{k\in\mathbb{Z}} \delta(t - t_k^O) \cdot (1 - x^{AxT}) - \beta^{AxT} \cdot x^{AxT} - \kappa^{AxT} \cdot \sum_{k\in\mathbb{Z}} \delta(t - t_k^L) \cdot x^{AxT}. \tag{3}$$

$$\tag{4}$$

Informally, the steady-state response of x^{AxT} amounts to

$$x^{AxT} = \frac{\alpha^{AxT} \cdot \sum_{k\in\mathbb{Z}} \delta(t - t_k^O)}{\alpha^{AxT} \cdot \sum_{k\in\mathbb{Z}} \delta(t - t_k^O) + \beta^{AxT} + \kappa^{AxT} \cdot \sum_{k\in\mathbb{Z}} \delta(t - t_k^L)}, \tag{5}$$

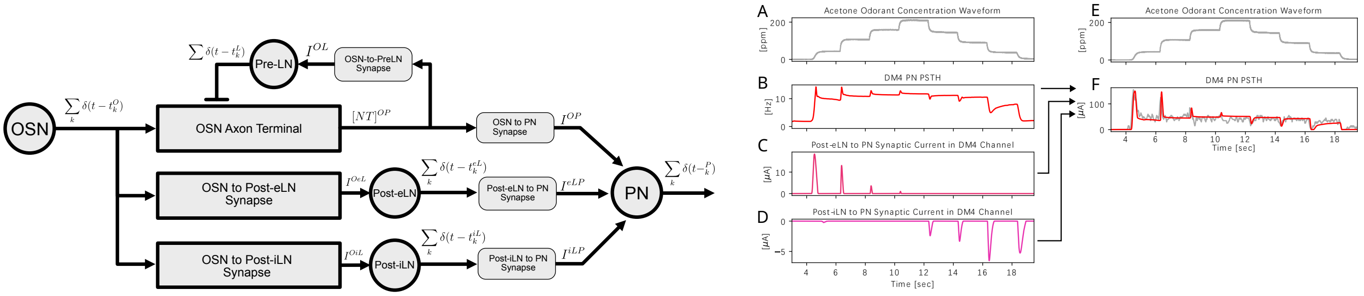

Figure S1: Single-channel Antennal Lobe circuit with temporal feedback. (left) Schematic diagram of the single-channel AL circuit with temporal Pre-LN feedback. The channel consists of 3 DNPs: a feedback DNP acting presynaptically on the OSN axon terminal (top branch), a feedforward DNP with Post-eLN (middle branch) and a feedforward DNP with Post-iLN (bottom branch). Adapted from Lazar et al. (2023) under Creative Commons Attribution License. (right) Response of the single-channel AL circuit to a staircase acetone concentration waveform. **(A)** Staircase concentration waveform. **(B)** Synaptic current from OSN to PN. **(C)** Synaptic current from Post-eLN to PN. **(D)** Synaptic current from Post-iLN to PN. **(E)** Same as in (A). **(F)** DM4 PN response to the staircase concentration waveform. (grey) Electrophysiology recording of the activity of the DM4 PN (Kim et al., 2015) in response to the acetone staircase concentration waveform.

i.e., a family of sigmoids of the OSN input parametrized by the Pre-LN feedback.

Single-Channel Antennal Lobe Subcircuit with Feedforward Post-eLN

The OSN to Post-eLN Synapse with parameters $[\alpha_1^{eL}, \beta_1^{eL}, \kappa_1^{eL}, \alpha_2^{eL}, \beta_2^{eL}, \alpha_3^{eL}, \beta_3^{eL}, \overline{g}_{max}^{eL}, E^{eL}]$ is described by (see Figure S1 (middle left))

$$\frac{d}{dt}x^{eL} = \alpha_1^{eL} \cdot x_2^{eL} \cdot (1 - x^{eL}) - \beta_1^{eL} \cdot x^{eL} - \kappa_1^{eL} x_3^{eL} \cdot x^{eL} \tag{6}$$

$$\frac{d}{dt}x_2^{eL} = \alpha_2^{eL} \cdot \sum_{k\in\mathbb{Z}} \delta(t - t_k^O) \cdot (1 - x_2^{eL}) - \beta_2^{eL} \cdot x_2^{eL} \tag{7}$$

$$\frac{d}{dt}x_3^{eL} = \alpha_3^{eL} \cdot \sum_{k\in\mathbb{Z}} \delta(t - t_k^O) \cdot (1 - x_3^{eL}) - \beta_3^{eL} \cdot x_3^{eL} \tag{8}$$

$$I^{eL} = \overline{g}_{max}^{eL} \cdot x^{eL} \cdot (V^{eL} - E^{eL}), \tag{9}$$

where I^{eL} is the synaptic current injected into the Post-eLN BSG, V^{eL} is the Post-eLN membrane voltage and E^{eL} is the reversal potential of the synapse.

For the Post-eLN BSG model the noise variance is $(\sigma^{eL})^2 = 0$ and the spike train generated by the Post-eLN amounts to

$$\sum_{k\in\mathbb{Z}} \delta(t - t_k^{eL}) \leftarrow \text{NoisyConnorStevens}(I^{eL}; \sigma^{eL}). \tag{10}$$

Single-Channel Antennal Lobe Subcircuit with Feedforward Post-iLN

The OSN to Post-iLN synapse with parameters $[\alpha_1^{iL}, \beta_1^{iL}, \kappa_1^{iL}, \alpha_2^{iL}, \beta_2^{iL}, \alpha_3^{iL}, \beta_3^{iL}, \overline{g}_{max}^{iL}, E^{iL}]$ is described by (Figure S1 (bottom left))

$$\frac{d}{dt}x^{iL} = \alpha_1^{iL} \cdot x_2^{iL} \cdot (1 - x^{iL}) - \beta_1^{iL} \cdot x^{iL} - \kappa_1^{iL} x_3^{iL} \cdot x^{iL} \tag{11}$$

$$\frac{d}{dt}x_2^{iL} = \alpha_2^{iL} \cdot \sum_{k\in\mathbb{Z}} \delta(t - t_k^O) \cdot (1 - x_2^{iL}) - \beta_2^{iL} \cdot x_2^{iL} \tag{12}$$

$$\frac{d}{dt}x_3^{iL} = \alpha_3^{iL} \cdot \sum_{k\in\mathbb{Z}} \delta(t - t_k^O) \cdot (1 - x_3^{iL}) - \beta_3^{iL} \cdot x_3^{iL} \tag{13}$$

$$I^{iL} = \overline{g}_{max}^{iL} \cdot x^{iL} \cdot (V^{iL} - E^{iL}). \tag{14}$$

where I^{iL} is the synaptic current injected into the Post-iLN BSG, V^{iL} is the Post-LN membrane voltage and E^{iL} is the reversal potential of the synapse.

For the Post-iLN BSG model the noise variance is $(\sigma^{iL})^2 = 0$ and the spike train generated by the Post-iLN amounts to

$$\sum_{k\in\mathbb{Z}} \delta(t - t_k^{iL}) \leftarrow \text{NoisyConnorStevens}(I^{iL}; \sigma^{iL}). \tag{15}$$

Single-Channel Antennal Lobe Circuit Current Injected into a Projection Neuron

In what follows we evaluate the current inject into a PN by the OSN to PN, Post-eLN to PN and the Post-iLN to PN synapses.

The OSN to PN synapse with parameters $[\alpha^{OP}, \beta^{OP}, \overline{g}_{max}^{OP}, E^{OP}]$ is described by the eqs. (Figure S1 (top left))

$$\frac{d}{dt}x^{OP} = \alpha^{OP} \cdot [NT]^{OP} \cdot (1 - x^{OP}) - \beta^{OP} \cdot x^{OP} \tag{16}$$

$$I^{OP} = \overline{g}_{max}^{OP} \cdot x^{OP} \cdot (V^P - E^{OP}), \tag{17}$$

where I^{OP} is the synaptic current injected into the PN BSG, V^P is the PN membrane voltage and E^{OP} is the reversal potential of the synapse.

The Post-eLN to PN synapse with parameters $[\alpha^{eLP}, \beta^{eLP}, \overline{g}_{max}^{eLP}, E^{eLP}]$ is described by the eqs. (Figure S1 (middle left))

$$\frac{d}{dt}x^{eLP} = \alpha^{eLP} \cdot \sum_{k\in\mathbb{Z}} \delta(t - t_k^{eL}) \cdot (1 - x^{eLP}) - \beta^{eLP} \cdot x^{eLP} \tag{18}$$

$$I^{eLP} = \overline{g}_{max}^{eLP} \cdot x^{eLP} \cdot (V^P - E^{eLP}). \tag{19}$$

where I^{eLP} is the synaptic current injected into the PN BSG, V^P is the PN membrane voltage and E^{eLP} is the reversal potential of the synapse.

The Post-iLN to PN synapse with parameters $[\alpha^{iLP}, \beta^{iLP}, \overline{g}_{max}^{iLP}, E^{iLP}]$ is described by the eqs. (Figure S1 (bottom left))

$$\frac{d}{dt}x^{iLP} = \alpha^{iLP} \cdot \sum_{k\in\mathbb{Z}} \delta(t - t_k^{iL}) \cdot (1 - x^{iLP}) - \beta^{iLP} \cdot x^{iLP} \tag{20}$$

$$I^{iLP} = \overline{g}_{max}^{iLP} \cdot x^{iLP} \cdot (V^P - E^{iLP}), \tag{21}$$

where I^{iLP} is the synaptic current injected into the PN BSG, V^P is the PN membrane voltage and E^{iLP} is the reversal potential of the synapse.

The Spike Train Generated at the Output of a Projection Neuron and Its Biological Validation

For the PN BSG model the noise variance is $(\sigma^P) = 0.00138493^2$ and v^P

$$v^P(t) = \sum_{k\in\mathbb{Z}} \delta(t - t_k^P) \leftarrow \text{NoisyConnorStevens}(I^{OP} + I^{eLP} + I^{iLP}; \sigma^P). \tag{22}$$

An example evaluation of the single channel circuit model in Figure S1(left) is shown in the same figure on the (right) The odorant concentration waveform and Pre-LN, Post-eLN and Post-iLN to PN synaptic currents estimated from physiology recording data are shown in grey. Response of each DNP (and DNP-BSG cascade) is shown in red. In Figure S1(top right) the same acetone staircase concentration waveform is shown. Model DM4 PN PSTH (red) compared with the DM4 PN physiology recording (grey) is depicted at (bottom right). For more details, see Lazar et al. (2023).

1.2 I/O Modeling of the Multi-Channel AL with Spatio-Temporal Feedback

The model architecture of the multi-channel AL circuit is shown on the left in Figure S2 (Lazar et al., 2023). An evaluation of the input/output relationship of the multi-channel AL circuit is shown on the right. (Right [i,v]) Acetone staircase odorant concentration waveform. ([ii]) Post-eLN to PN synaptic currents. Different hues of red indicate strengths of synaptic current, with strongest due to the Post-eLN receiving input from the Or59b OSN indicated in dark red. (Right [iii]) Post-iLN to PN synaptic currents. Different hues of blue indicate strengths of synaptic current, with strongest due to the Post-iLN receiving input from the Or59b OSN indicated in dark blue. (Right[iv]) OSN Axon-Terminal to PN synaptic current shown as a heatmap. Different hues of grey indicate strengths of synaptic current. Refer to colorbar for scale. (Right [vi]) Multi-channel PN PSTH, with synaptic current of ON and OFF pathways along Or59b/DM4 channel shown as blue and red arrows, respectively.

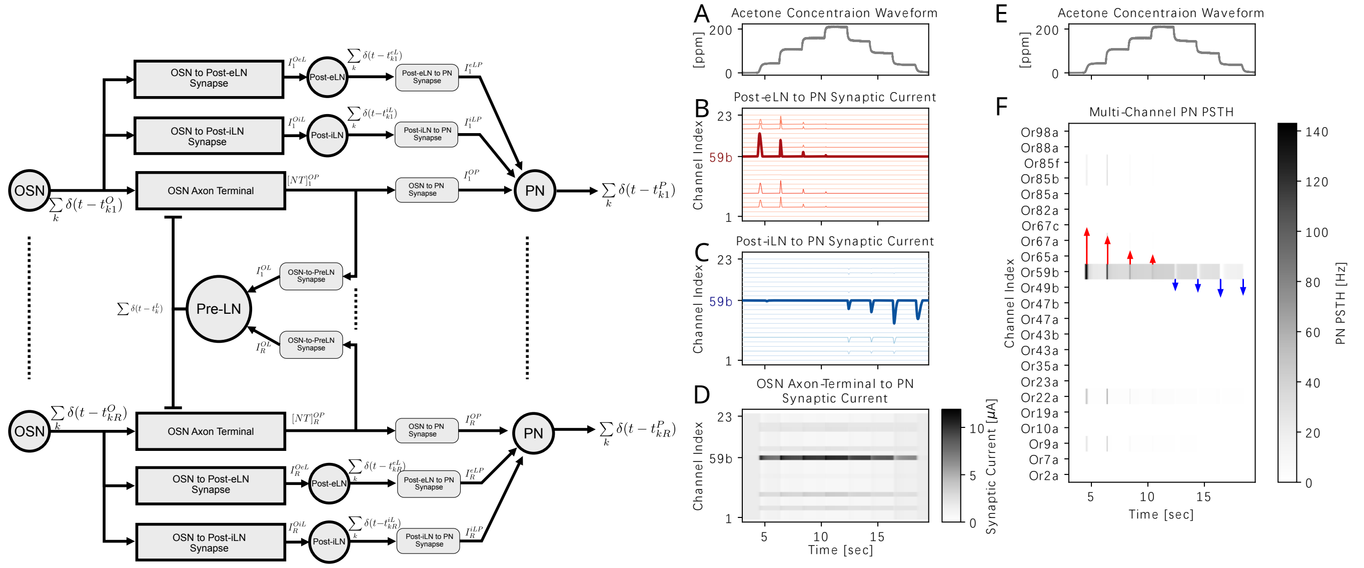

Figure S2: Multi-Channel Antennal Lobe circuit with spatio-temporal feedback. (left) Schematic diagram of the multi-channel AL circuit with spatio-temporal feedback. Adapted from Lazar et al. (2023) under Creative Commons Attribution License. (right) Response of the multi-channel AL circuit to a staircase acetone concentration waveform. **(A)** Staircase concentration waveform. **(B)** Synaptic currents from Post-eLNs to PNs in each of the 23 channels. **(C)** Synaptic currents from Post-iLNs to PNs in each of the 23 channels. **(D)** Synaptic currents from OSNs to PNs in each of the 23 channels. **(E)** Same as in (A). **(F)** PN responses to the staircase acetone concentration waveform in each channel.

Note that the results obtained in Figure S2 were obtained above and in Lazar et al. (2023) using simulations. Biological validation of the multi-channel Antennal Lobe is not yet available in the literature.

References

Kim, A. J., Lazar, A. A., and Slutskiy, Y. B. (2015). Projection neurons in drosophila antennal lobes signal the acceleration of odor concentrations. *eLife* , e06651

Lazar, A. A., Liu, T., and Yeh, C.-H. (2023). The functional logic of odor information processing in the drosophila antennal lobe. *PLOS Computational Biology* 19, 1–33. doi:10.1371/journal.pcbi.1011043. Doi: 10.1371/journal.pcbi.1011043

2 Additional Supplementary Figures

This section includes the following 7 supplementary figures:

- Figure S3: Evaluating the effect of the PN-KC connectivity parameter Q on the dendritic KC input for Diethyl Succinate.
- Figure S4: Evaluating the effect of the PN-KC connectivity parameter Q on the dendritic KC input for Ethyl Butyrate.
- Figure S4: Ranking of KC dendritic inputs is preserved across different instantiations of PN-KC bipartite graphs.
- Figure S6: Response of the OEM to 110 odorants in the DoOR database.
- Figure S7: Odorant semantics information of a mixture of Methanol and Benzyl Alcohol with different constant concentration amplitude ratios encoded in the time domain across the population of KCs.
- Figure S8: Odorant semantics information of a mixture of Acetone and Diethyl Succinate with different constant concentration amplitude ratios encoded in the time domain across the population of KCs.
- Figure S9: Odorant semantics information of a mixture of Acetone and 2,3-Butanedione with different constant concentration amplitude ratios encoded in the time domain across the population of KCs.

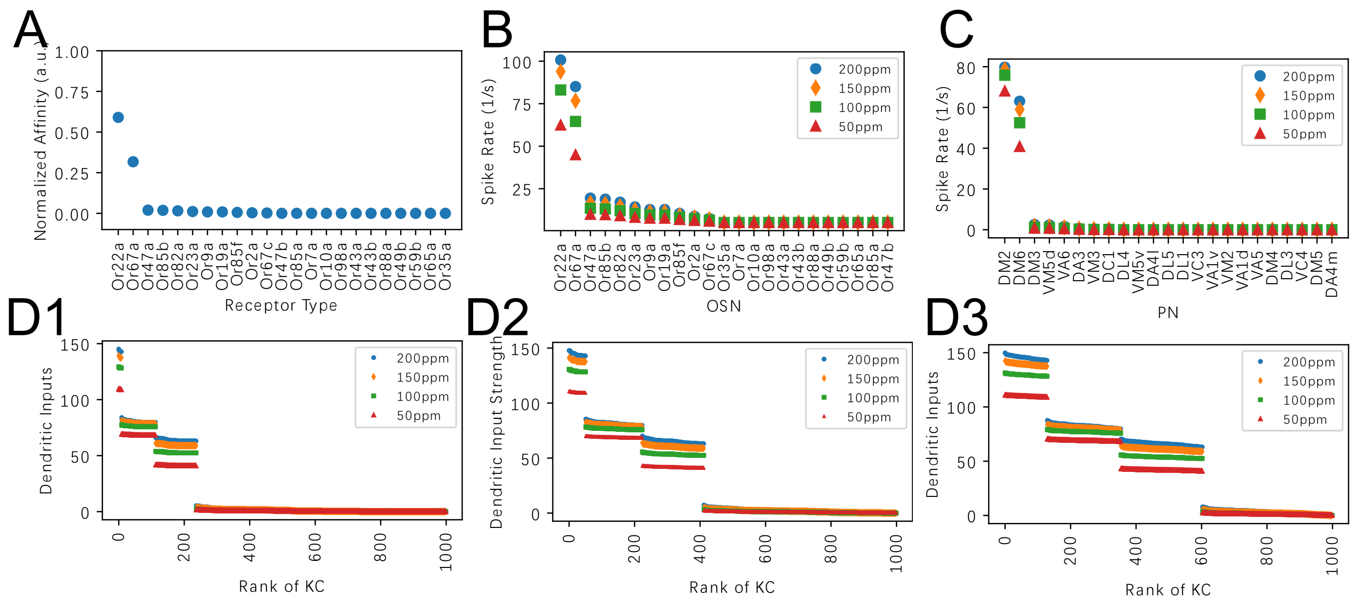

Figure S3: Evaluating the effect of the PN-KC connectivity parameter Q on the dendritic KC input for Diethyl Succinate. **(A)** Diethyl Succinate affinity in descending order. Affinity is normalized by the sum of all affinity values across receptor types. **(B)** Steady-state responses of OSNs to Diethyl Succinate at 4 different constant amplitude concentration levels. **(C)** Steady-state responses of PNs to Diethyl Succinate at different constant concentration levels. **(D)** Dendritic inputs to each KC in descending order of input strength, at 4 different constant concentration levels. **(D1)** Q = 3, **(D2)** Q = 6, **(D3)** Q = 9. The horizontal axis lists the KCs in ranking order.

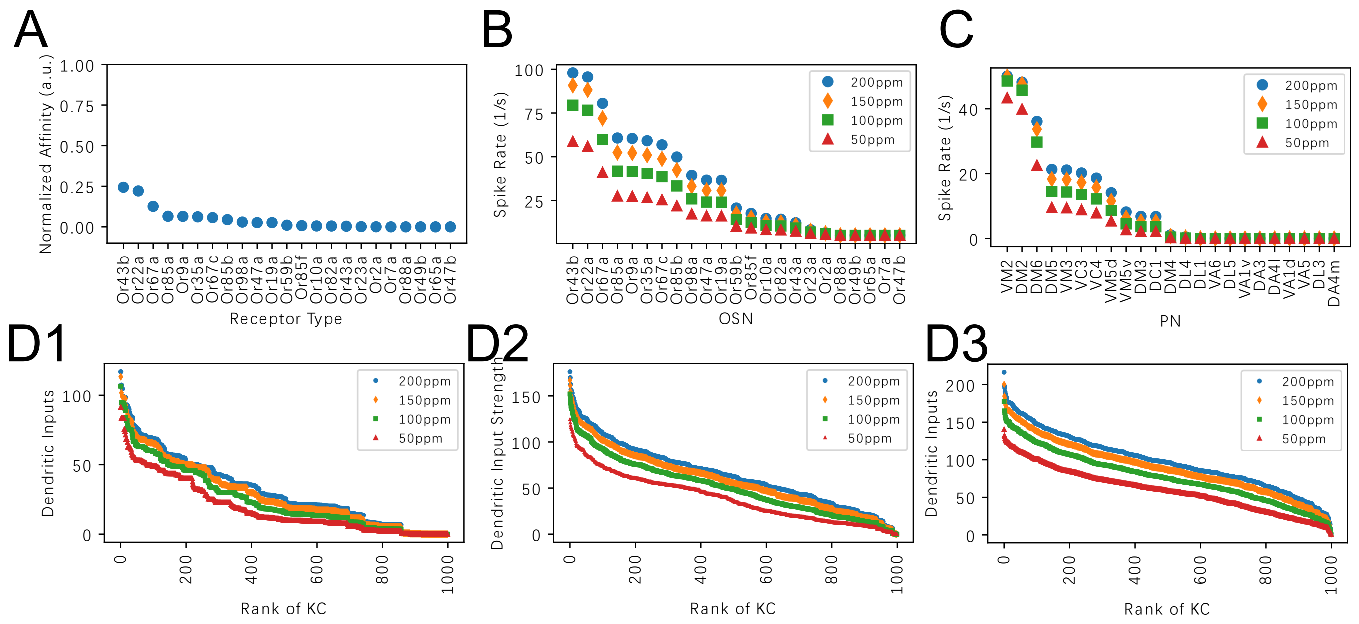

Figure S4: Evaluating the effect of the PN-KC connectivity parameter Q on the dendritic KC input for Ethyl Butyrate. **(A)** Ethyl Butyrate affinity in descending order. Affinity is normalized by the sum of all affinity values across receptor types. **(B)** Steady-state responses of OSNs to Ethyl Butyrate at 4 different constant amplitude concentration levels. **(C)** Steady-state responses of PNs to Ethyl Butyrate at different constant concentration levels. **(D)** Dendritic inputs to each KC in descending order of input strength, at 4 different constant concentration levels. **(D1)** $Q = 3$, **(D2)** $Q = 6$, **(D3)** $Q = 9$. The horizontal axis lists the KCs in ranking order.

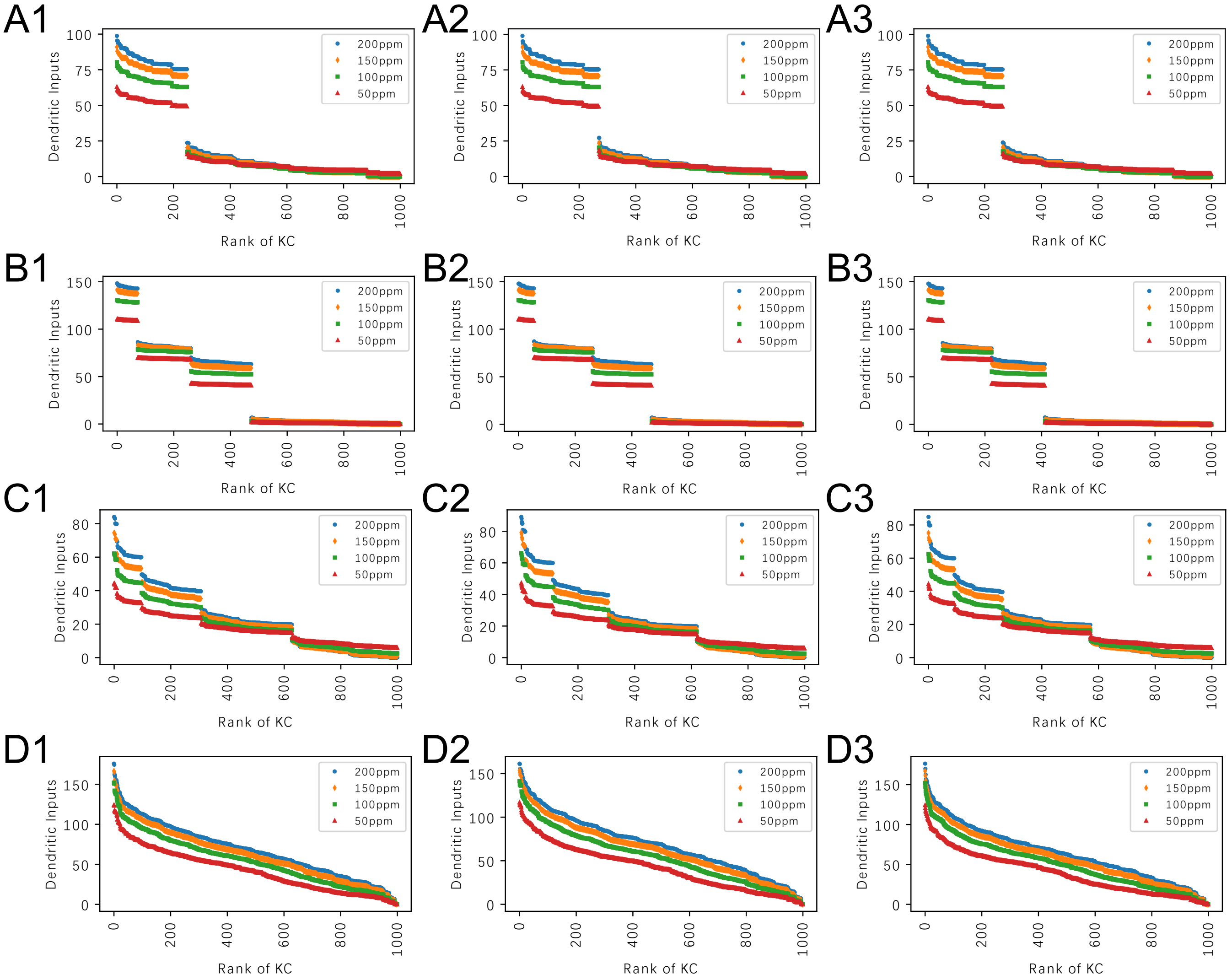

Figure S5: Ranking of KC dendritic inputs is preserved across different instantiations of PN-KC bipartite graphs. **(A)** Acetone. **(B)** Diethyl Succinate. **(C)** Nerol. **(D)** Ethyl Butyrate. The connectivity matrices of the PN-KC bipartite graphs in each of columns 1, 2 and 3 are instantiated using different random seeds. The parameter for the bipartite graph is $Q = 6$.

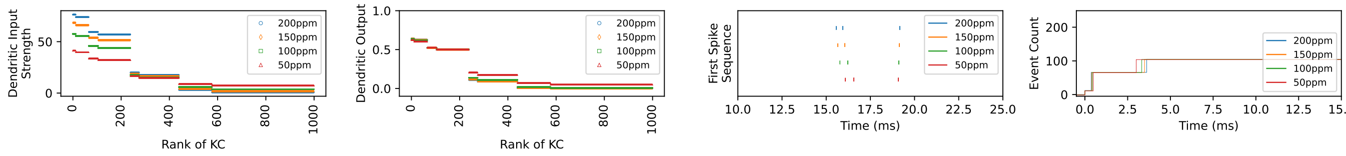

(1) Ethyl Cinnamate

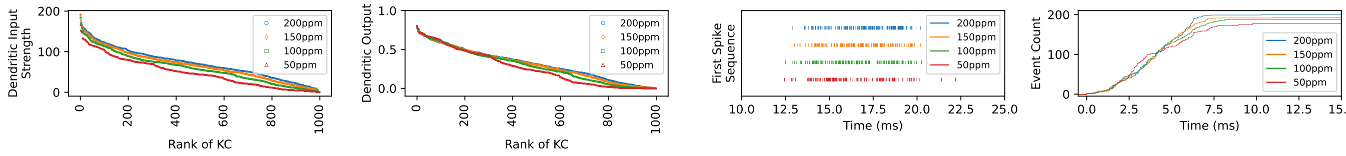

(2) E2-Hexenol

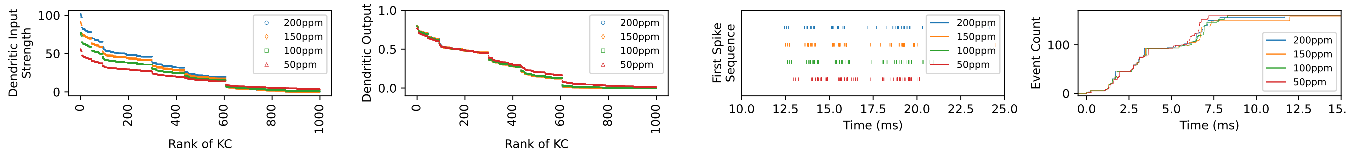

(3) α-Terpineol

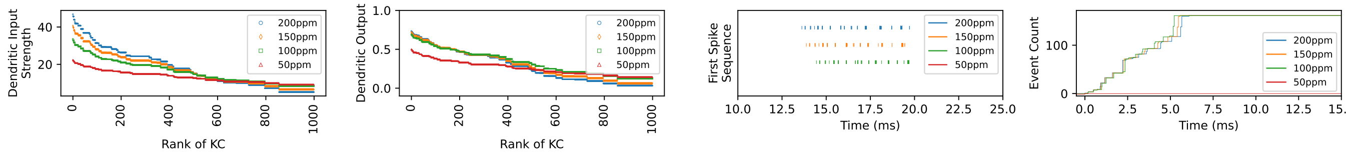

(4) Putrescine

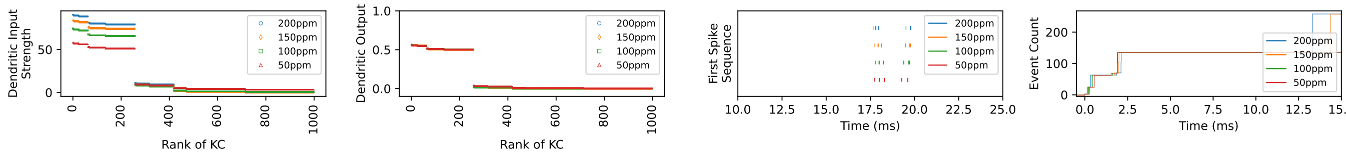

(5) Hexyl Hxanoate

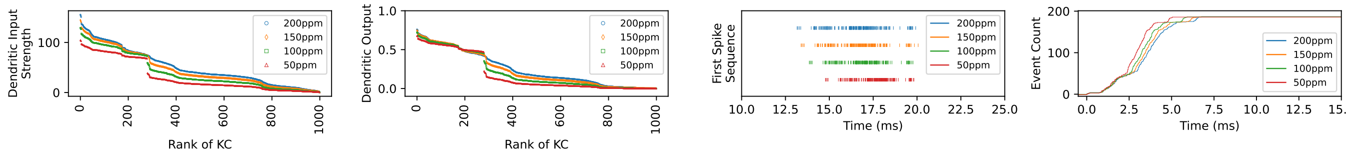

(6) 1-Penten-3-ol

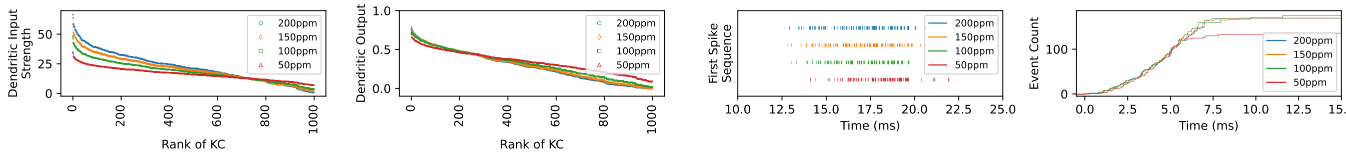

(7) Isobutyric Acid

Figure S6: Response of the OEM to 110 odorants in the DoOR database. **(1st column)** Ranking of KC dendritic inputs. **(2nd column)** Ranking of KC dendritic outputs. **(3rd column)** Odorant semantics encoded in the time domain across the population of KCs as the first spike sequence code. The first spikes of each of the active KCs in response to each odorant are collected onto a single row for each of the odorant concentration amplitude values. **(4th column)** The cumulative interspike intervals of the first spike sequence code in the third column.

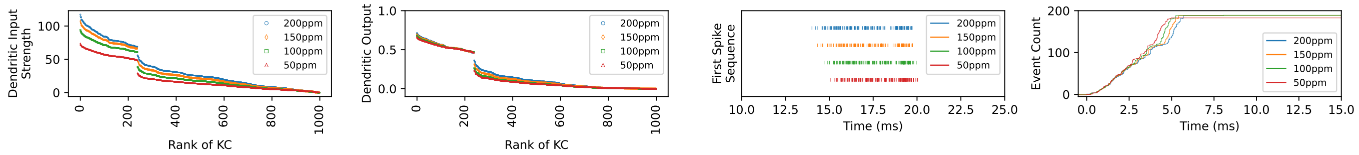

(8) Phenylacetaldehyde

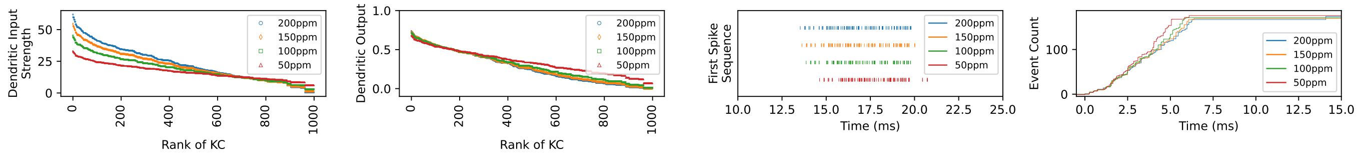

(9) α-Pinene

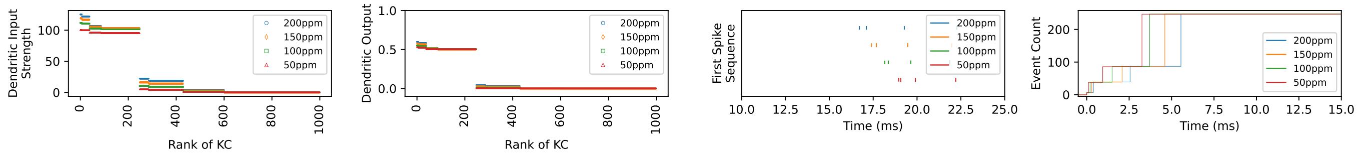

(10) Geranyl Acetate

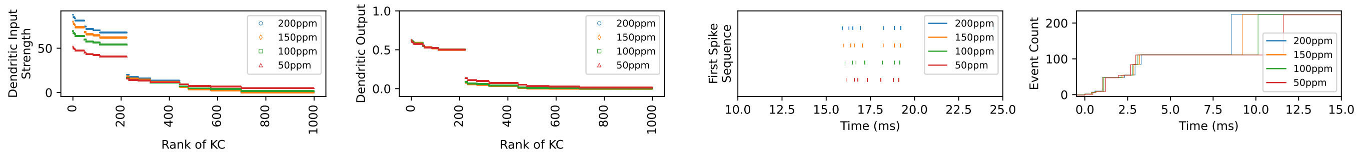

(11) Ethyl Octanoate

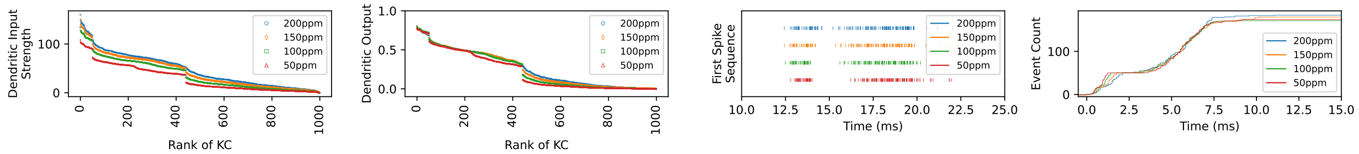

(12) Ethyl Propionate

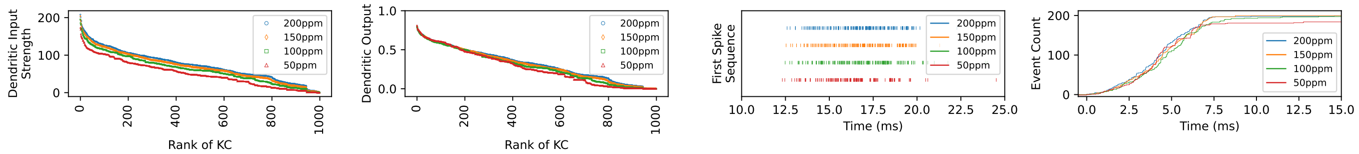

(13) Butyl Acetate

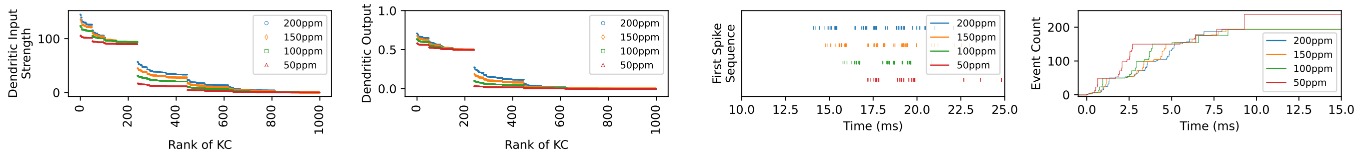

(14) Phenethyl Alcohol

Figure S6: Continued.

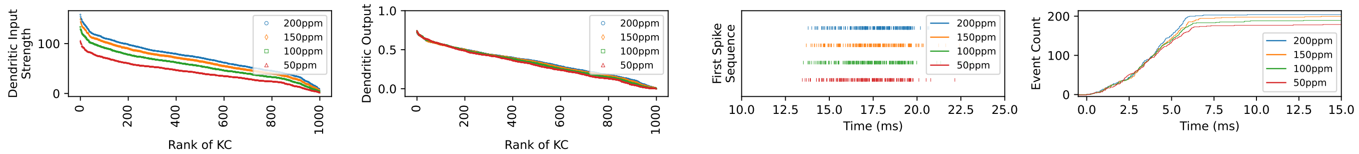

(15) 1-Pentanol

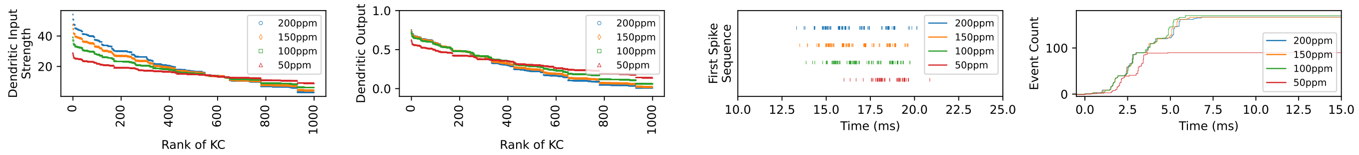

(16) Ammonium Hydroxid

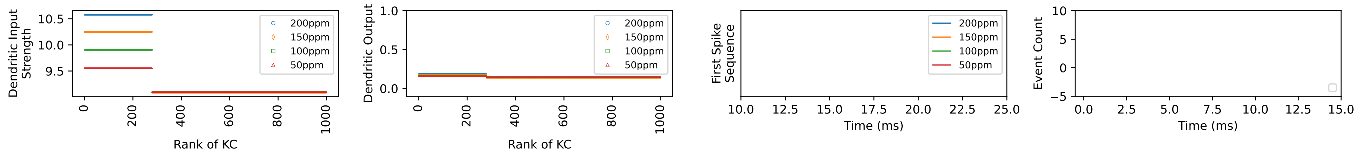

(17) Geraniollycerol

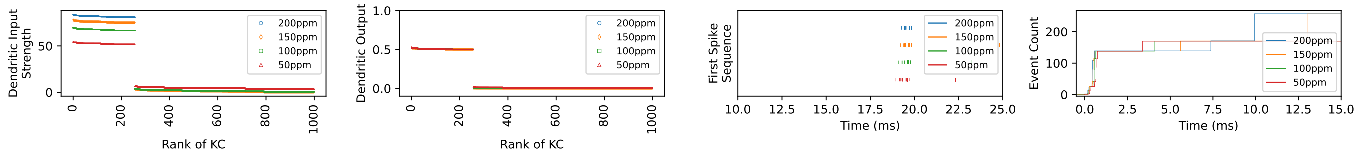

(18) (1S)-(+)-3-Carene

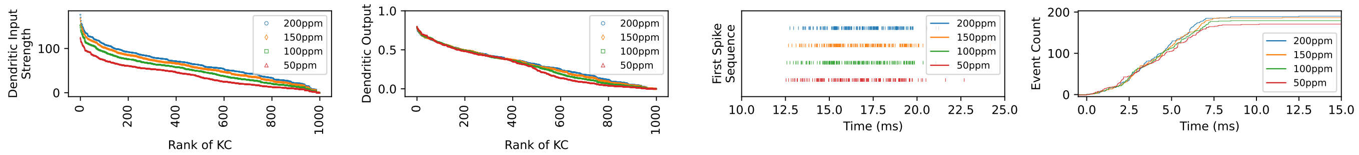

(19) Ethyl Butyrate

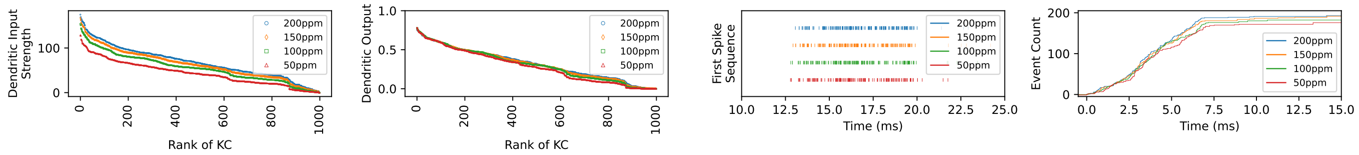

(20) Propyl Acetate

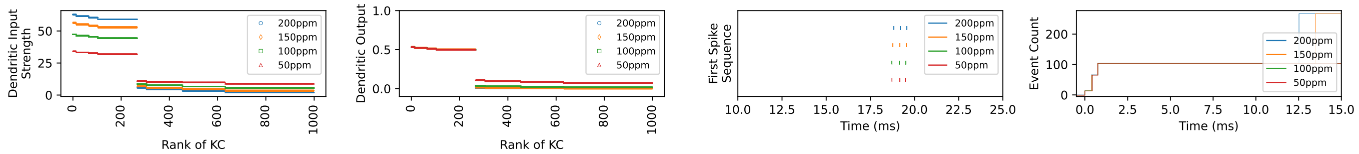

(21) Octanoic Acid

Figure S6: Continued.

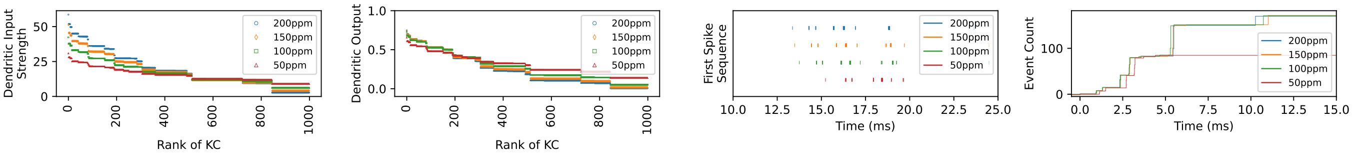

(22) β-Myrcene

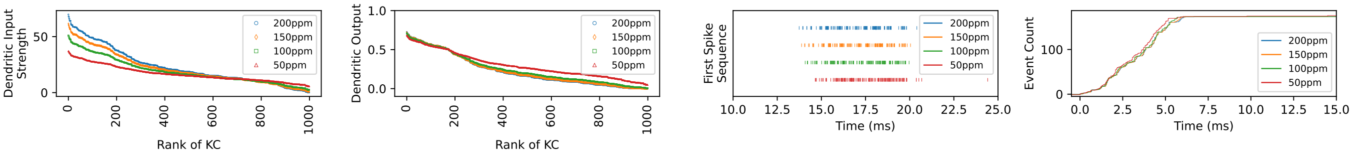

(23) Ethanol

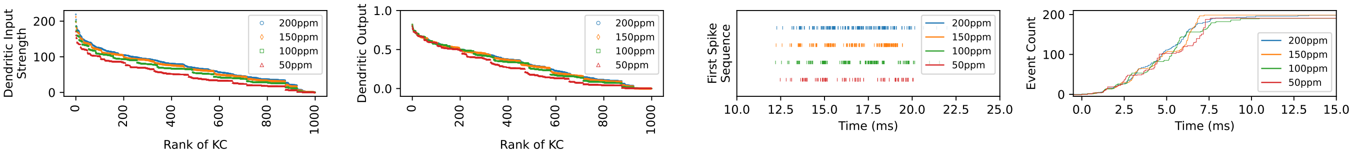

(24) Isobutyl Acetate

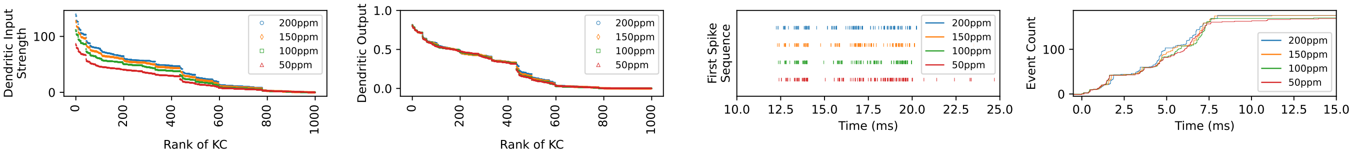

(25) Linalool

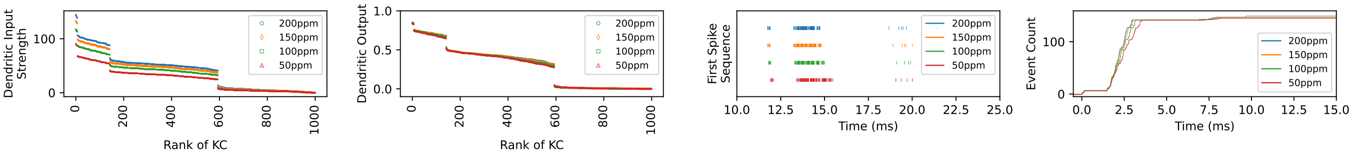

(26) Butyric Acid

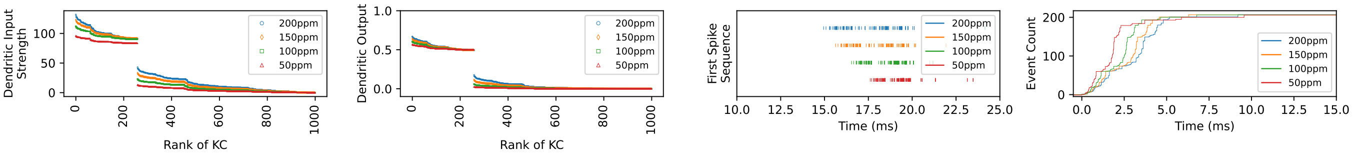

(27) 1-Octanol

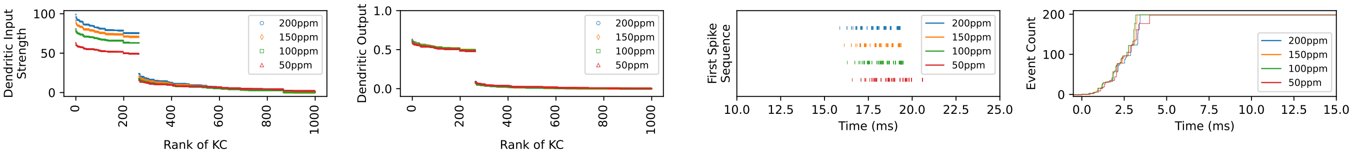

(28) Acetone

Figure S6: Continued.

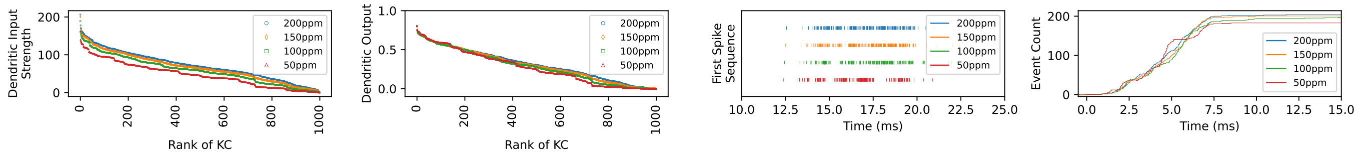

(29) Ethyl 3-Hydroxybutyrate

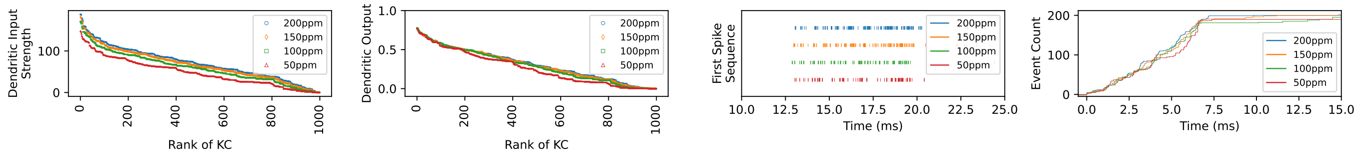

(30) Pentyl Acetate

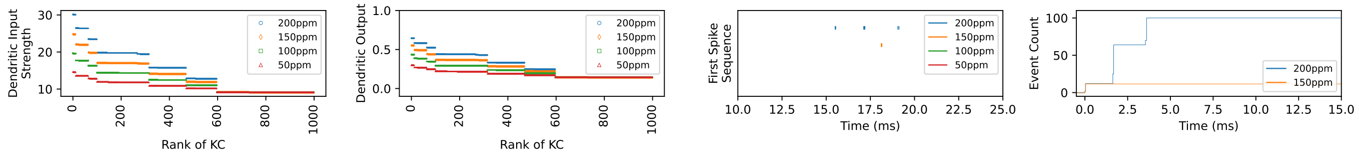

(31) α-Humulene

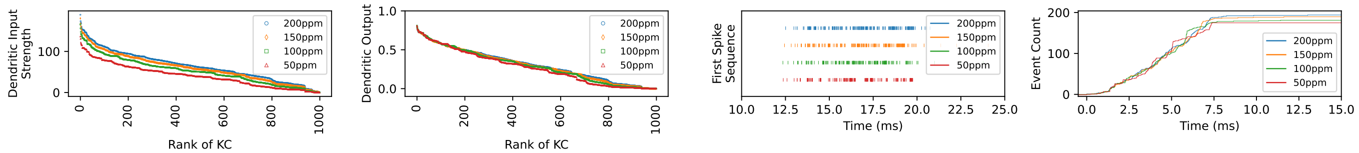

(32) 2-Heptanone

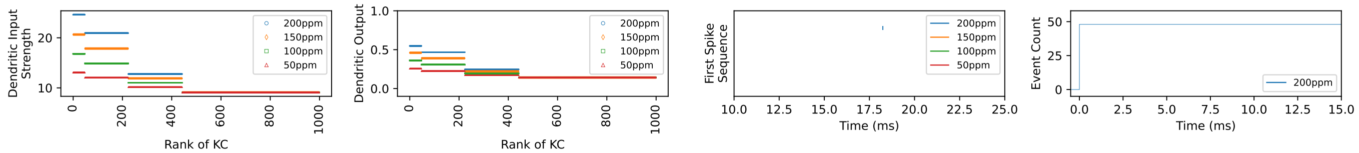

(33) γ-Pecalactone

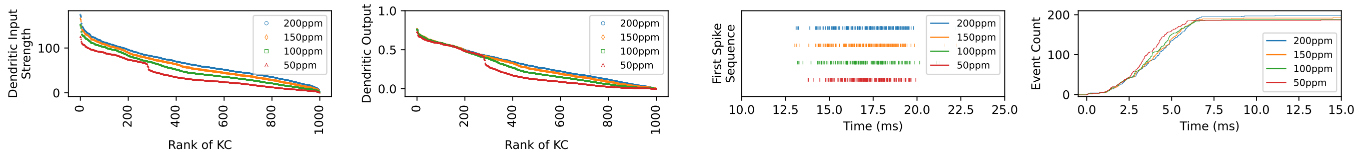

(34) 2-Pentanol

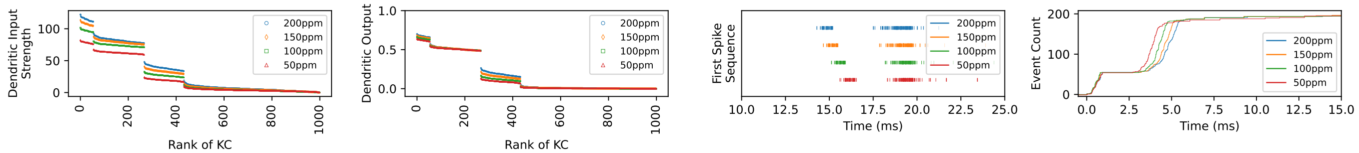

(35) γ-Octalactone

Figure S6: Continued.

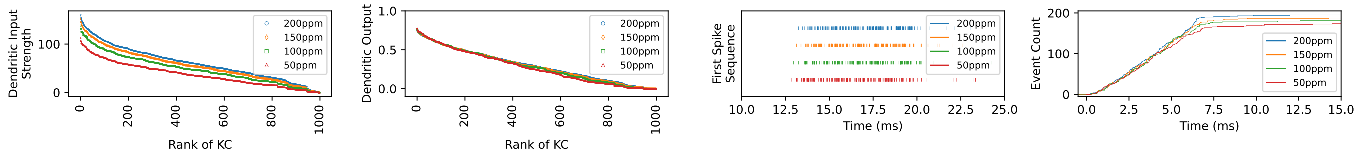

(36) 1-Octen-3-ol

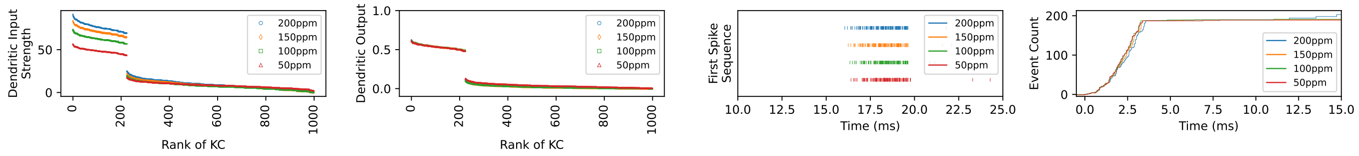

(37) Hexanoic Acid

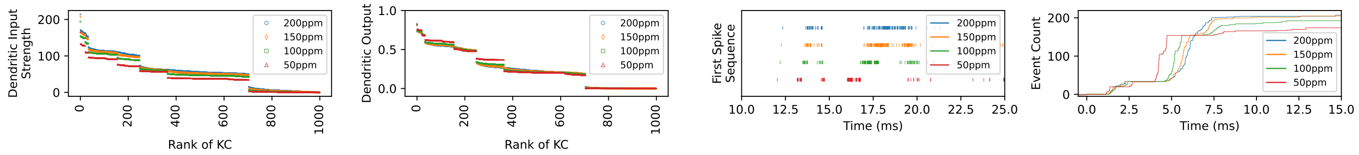

(38) E2-Hexenyl Acetate

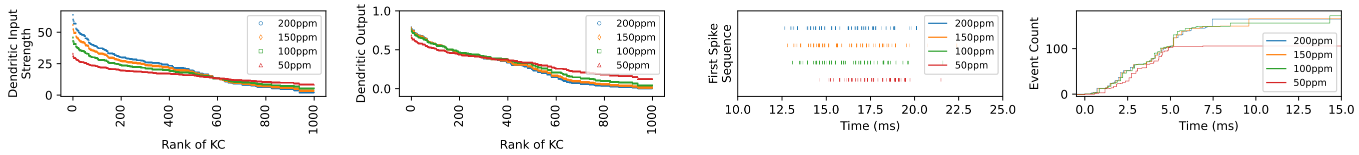

(39) Lactic Acid

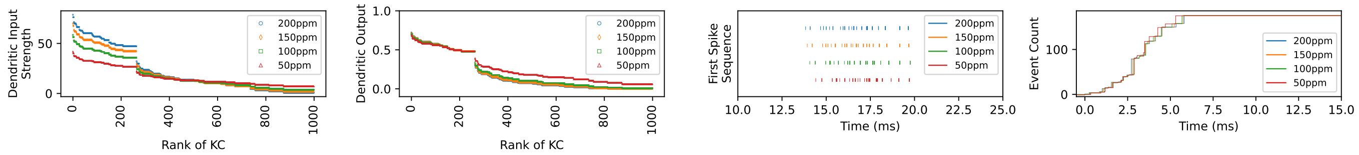

(40) Dimethyl Sulfide

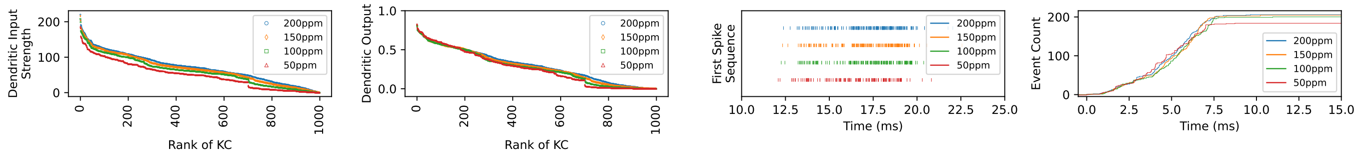

(41) Isopentyl Acetate

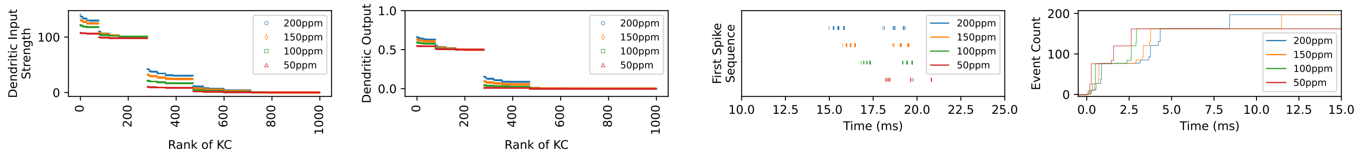

(42) 2,3-Butanediol

Figure S6: Continued.

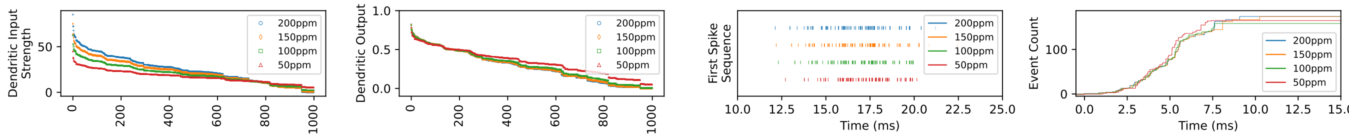

(43) Propionic Acid

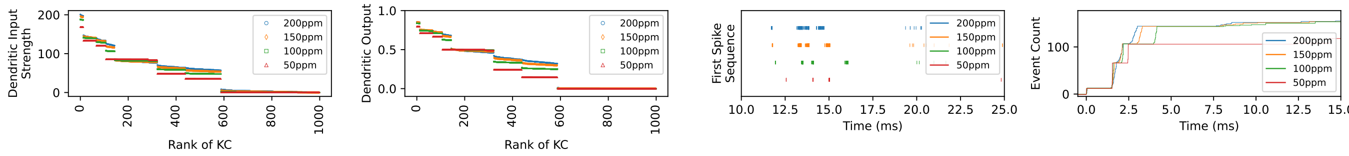

(44) Methyl Benzoate

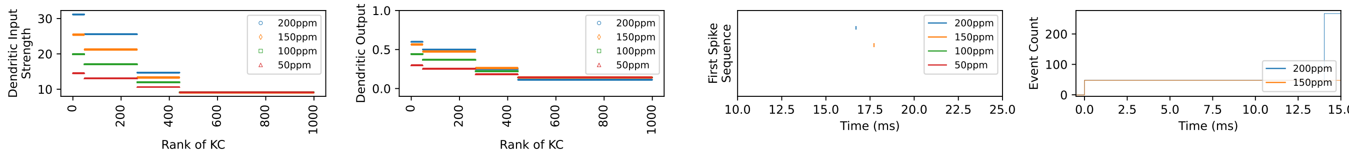

(45) δ-Decalactone

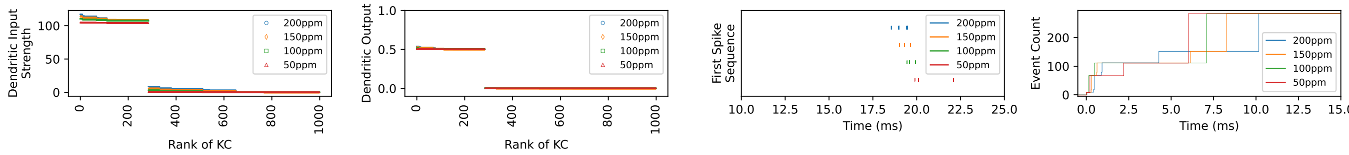

(46) Methyl Salicylate

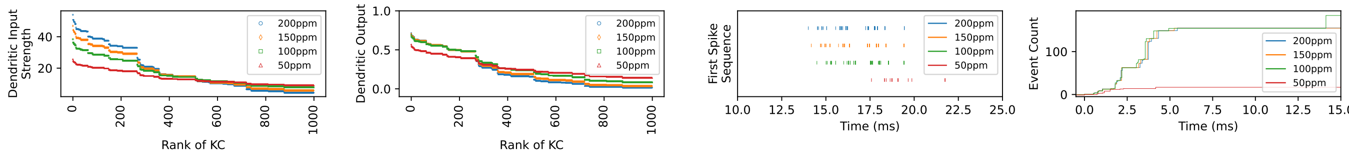

(47) Heptanoic Acid

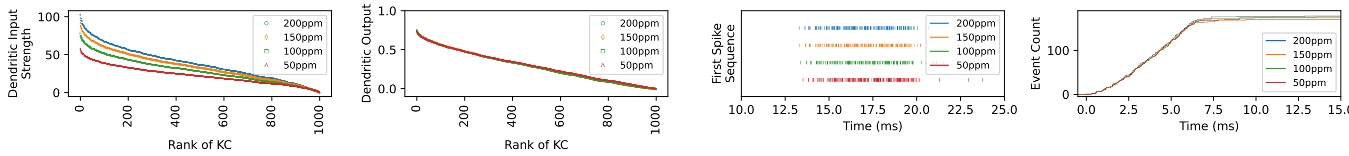

(48) 2,3-Butanedione

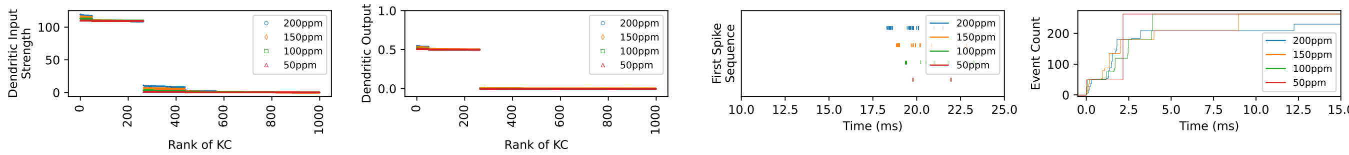

(49) Methyl Acetate

Figure S6: Continued.

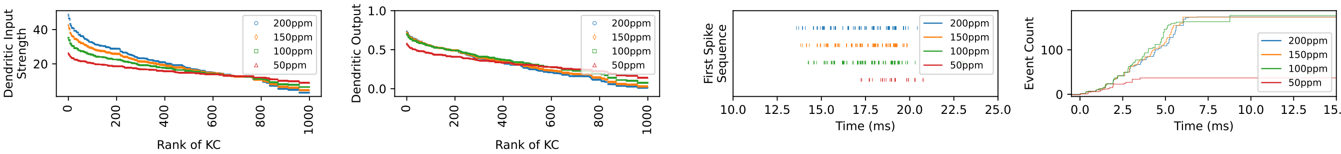

(50) Cadaverine

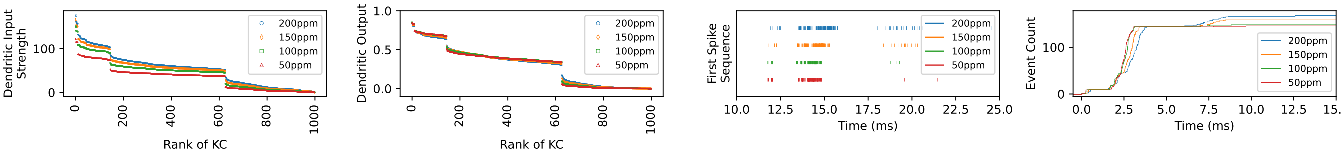

(51) Furfural

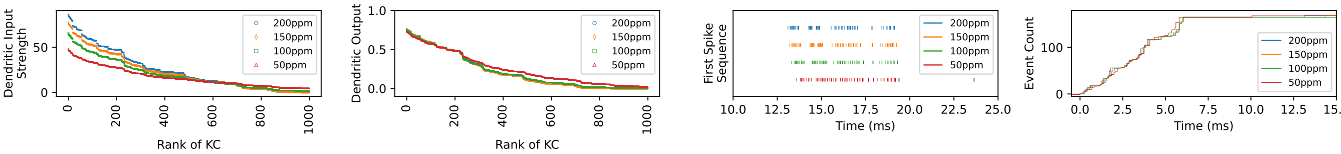

(52) Terpinolene

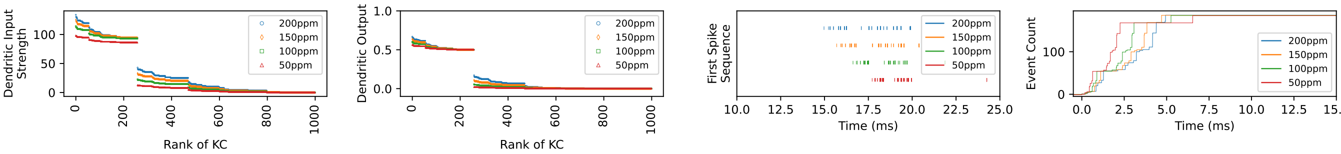

(53) Hexyl Butyrate

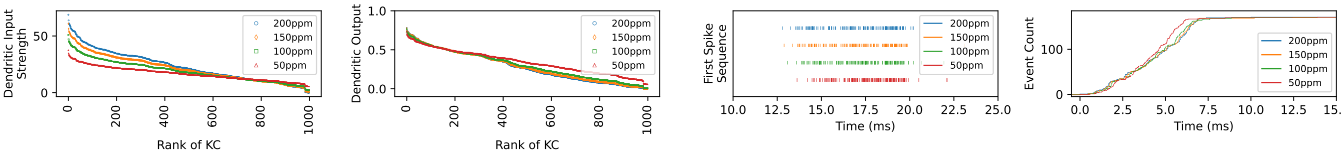

(54) Isopentanoic Acid

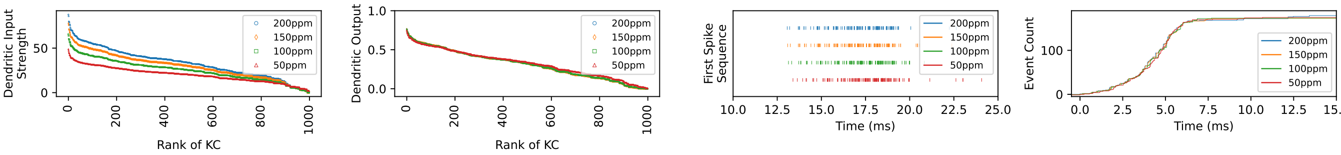

(55) Butanal

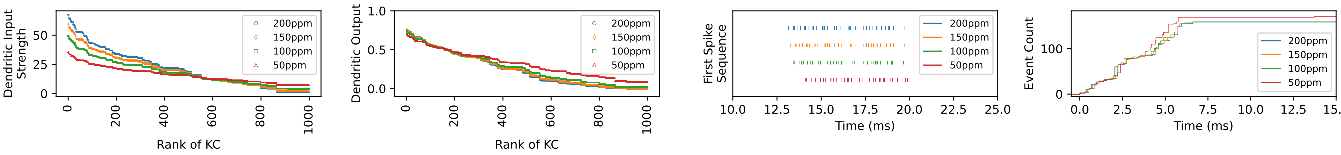

(56) β-Citronellol

Figure S6: Continued.

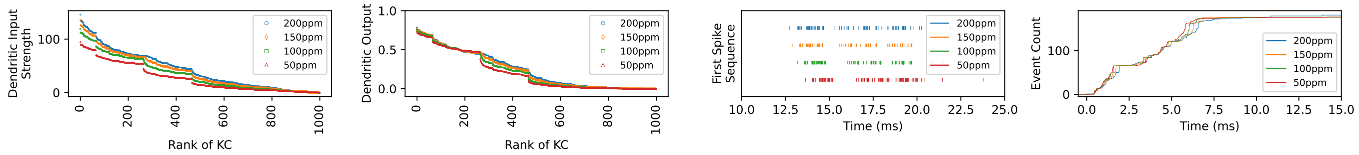

(57) Ethyl Acetate

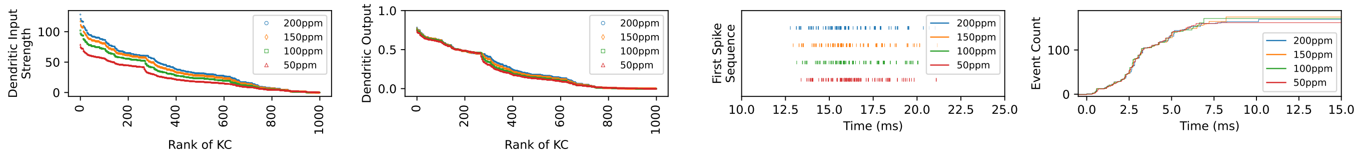

(58) 2-Butanone

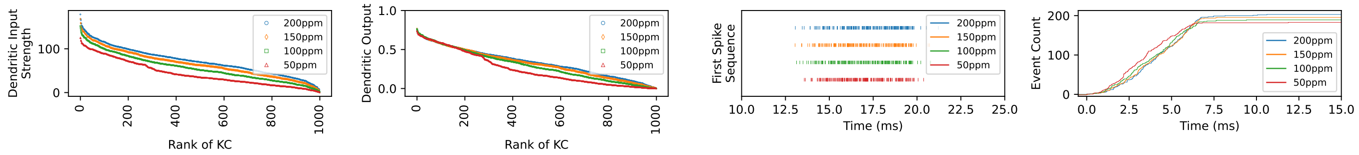

(59) 1-Hexanol

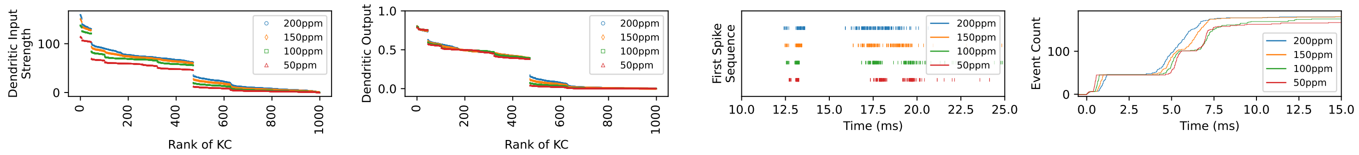

(60) 3-Methylthio-1-propanol

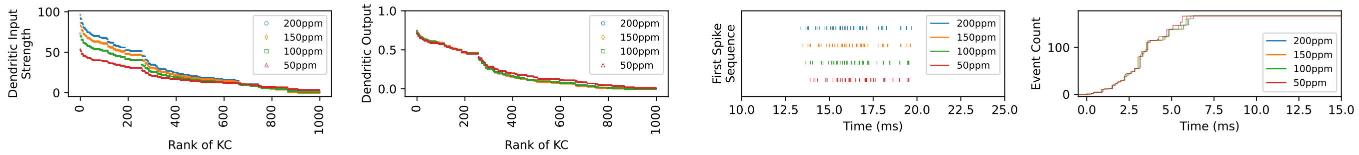

(61) Limonene

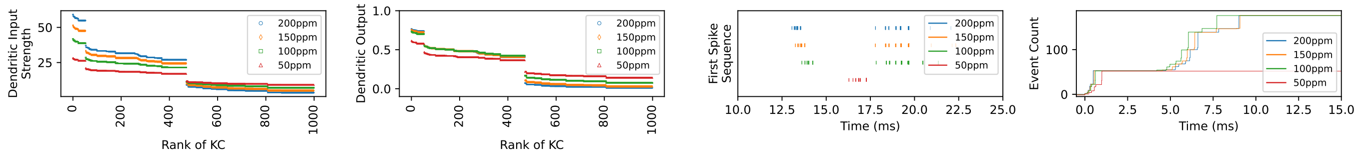

(62) β-Pinene

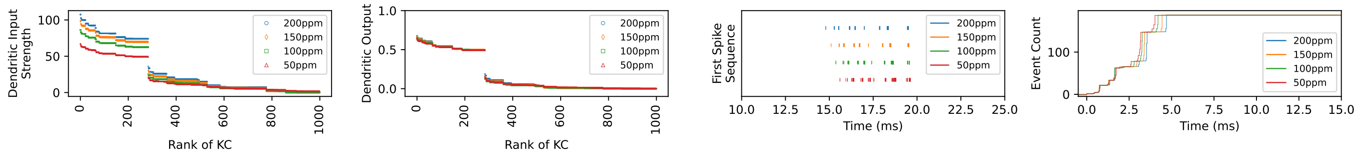

(63) Linoleic Acid

Figure S6: Continued.

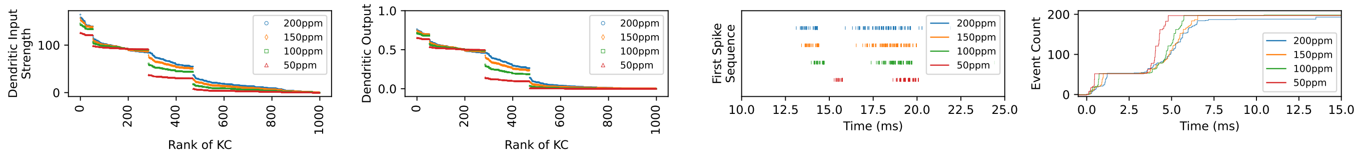

(64) Acetophenone

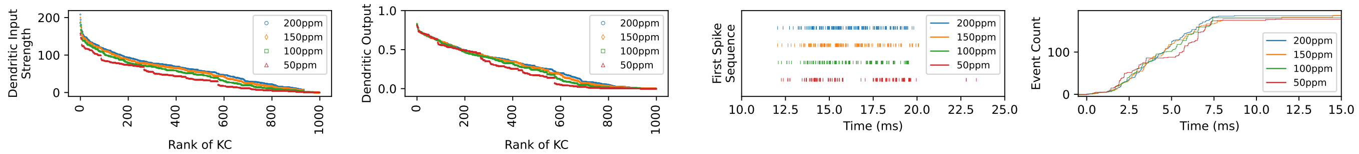

(65) Ethyl Trans-2-butenoate

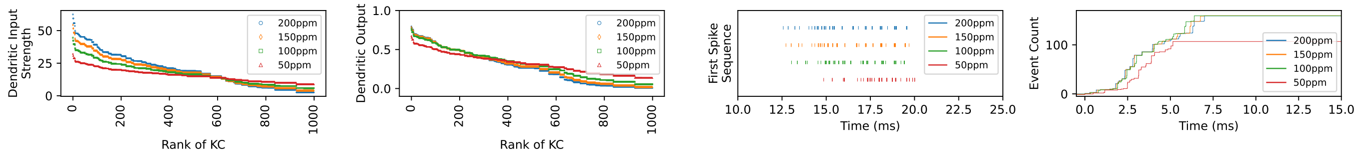

(66) Methanoic Acid

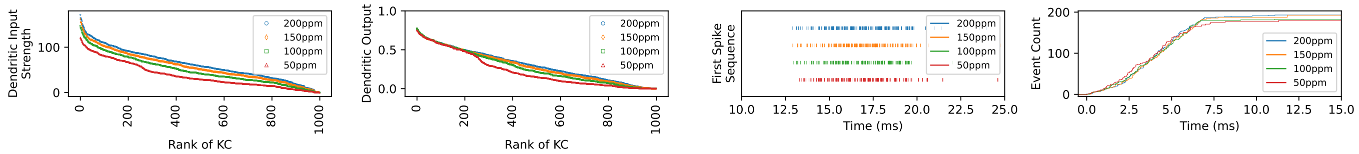

(67) Methyl Butyrate

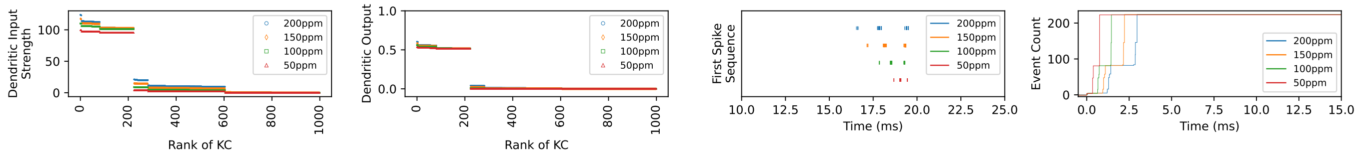

(68) Methyl Octanoate

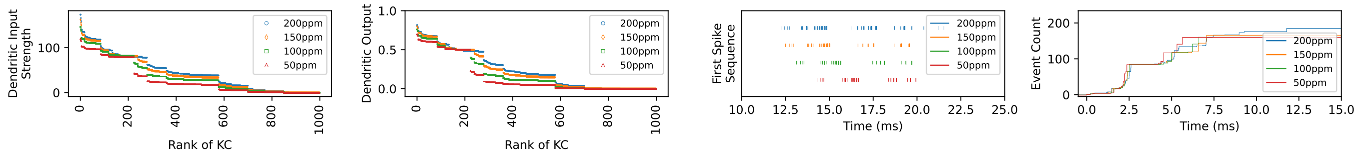

(69) Ethyl Hexanoate

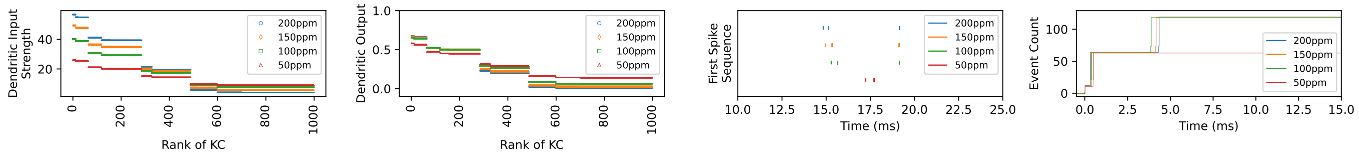

(70) Ethyl Decanoate

Figure S6: Continued.

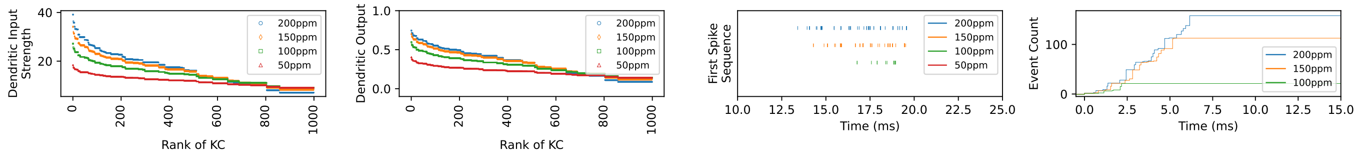

(71) p-Cymene

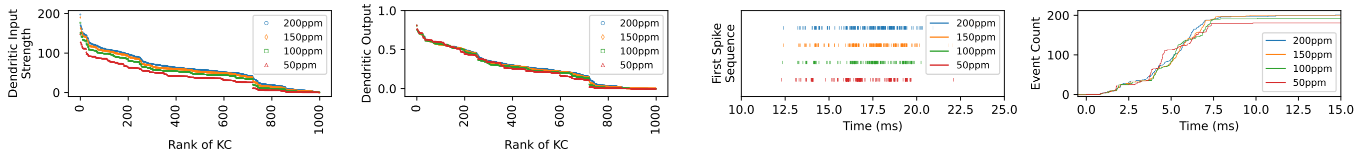

(72) 3-Methyl-2-buten-1-ol

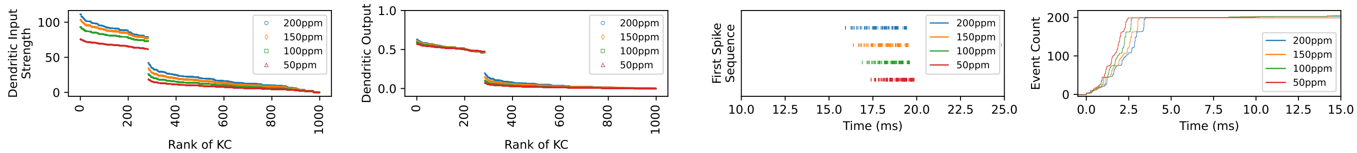

(73) 1-Propanol

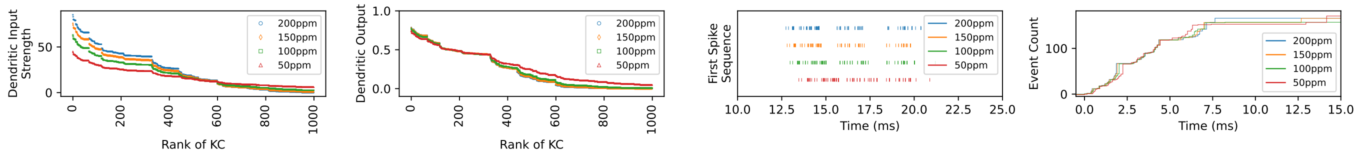

(74) Eugenol

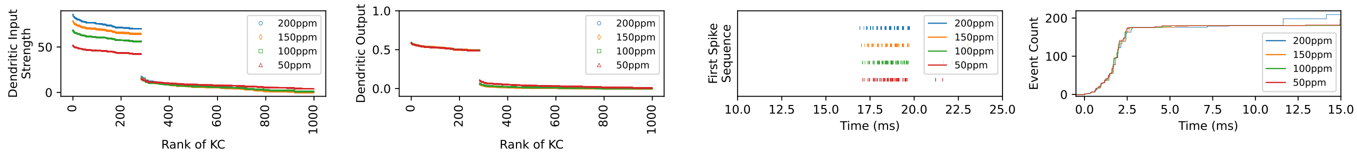

(75) Ethyl Methanoate

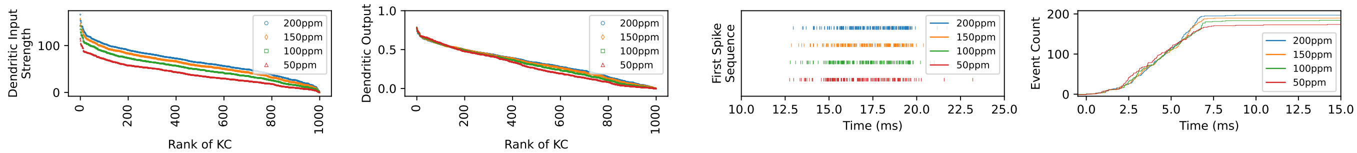

(76) Z2-Hexenol

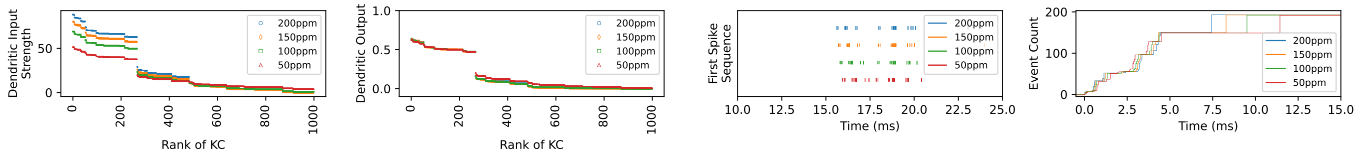

(77) 2-Ethylhexanoic Acid

Figure S6: Continued.

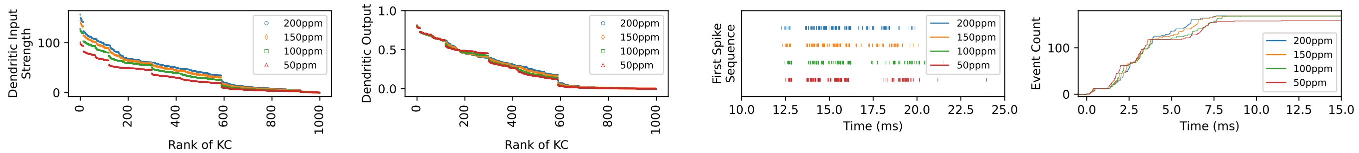

(78) γ-Hexalactone

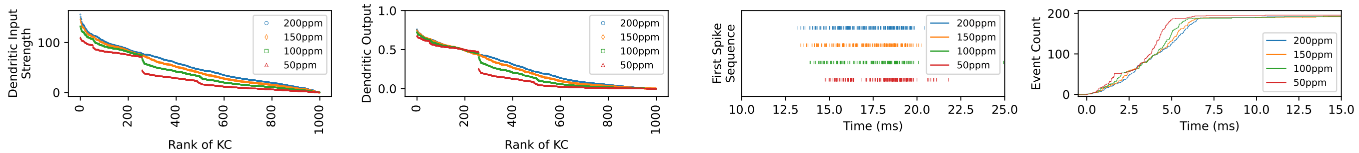

(79) 3-Methylbutanol

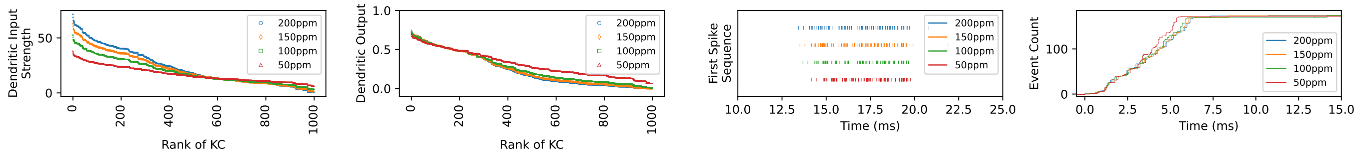

(80) Pyruvic Acid

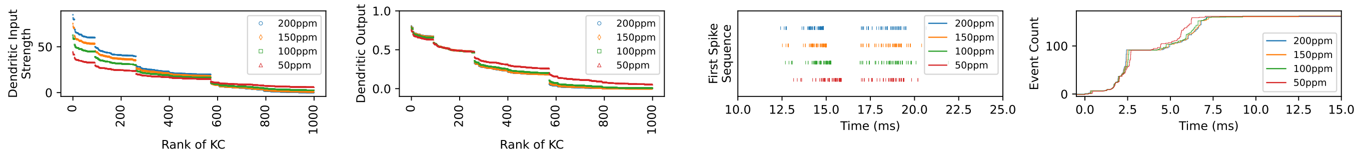

(81) Nerol

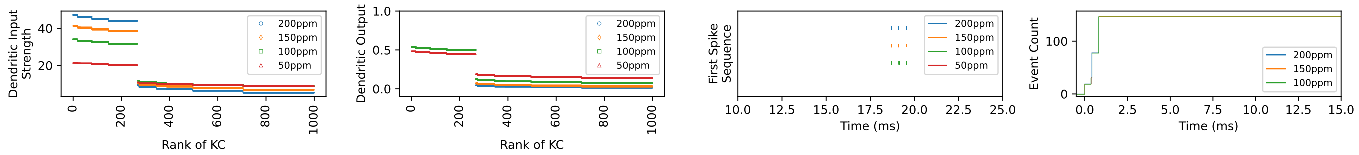

(82) Nonanoic Acid

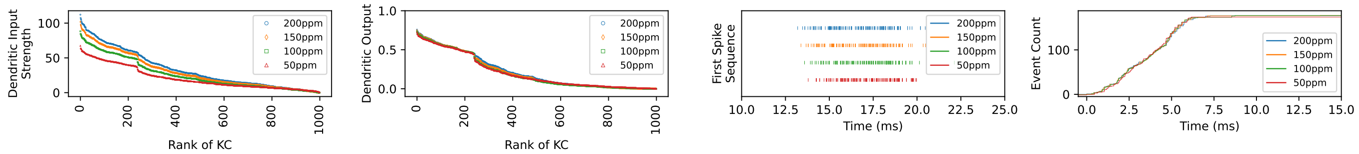

(83) γ-butyrolactone

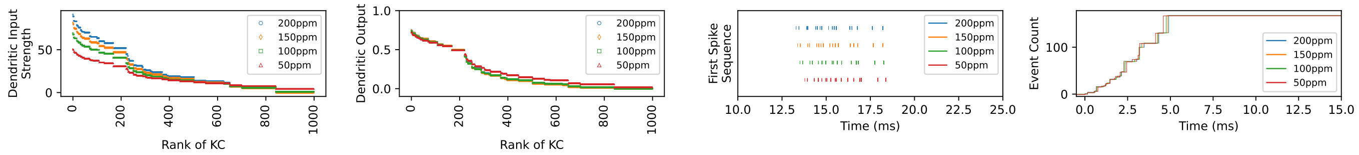

(84) Geraniol

Figure S6: Continued.

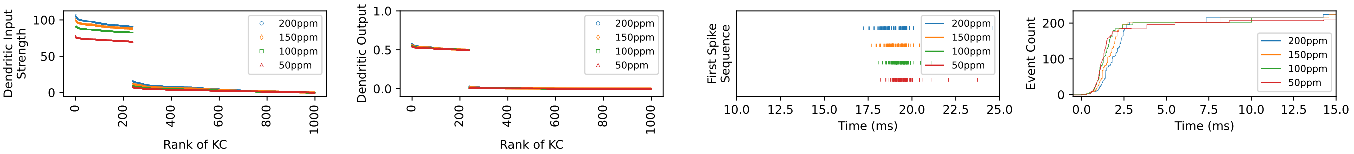

(85) Phenethyl Acetate

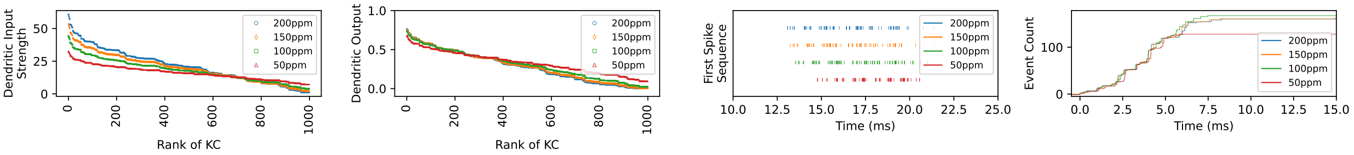

(86) 2-Propenal

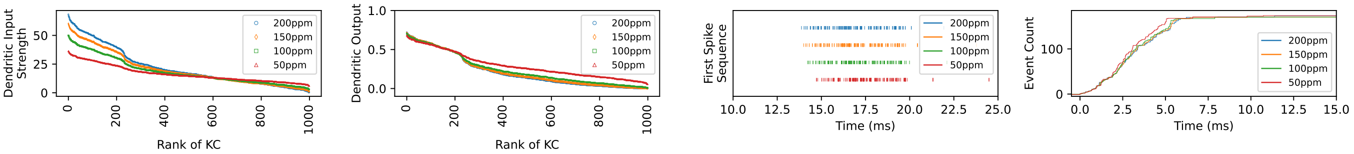

(87) Methanol

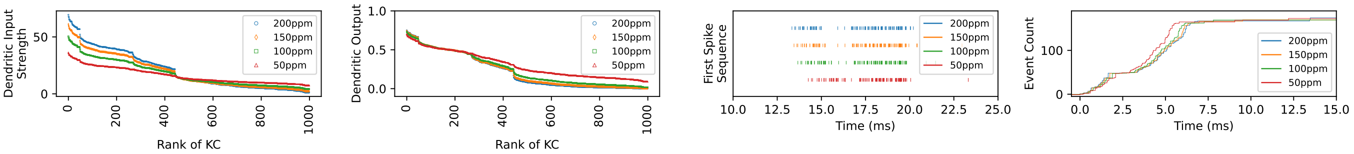

(88) Propanal

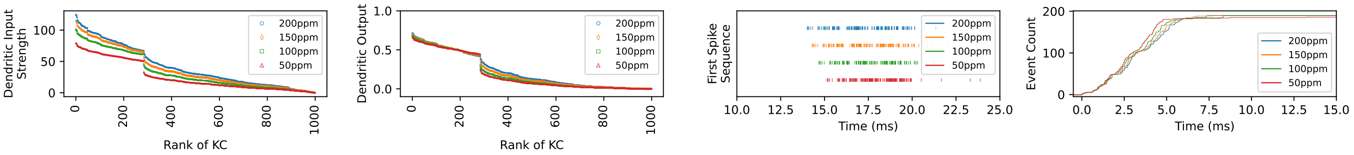

(89) Acetaldehyde

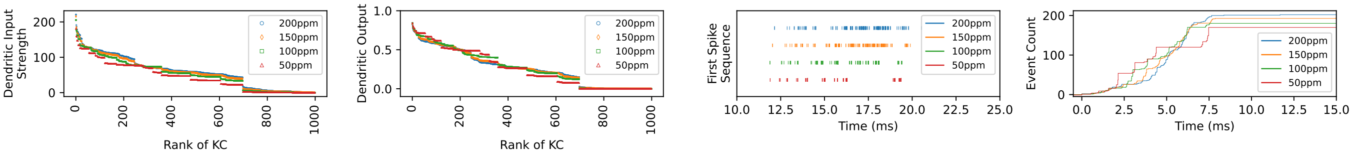

(90) Methyl Hexanoate

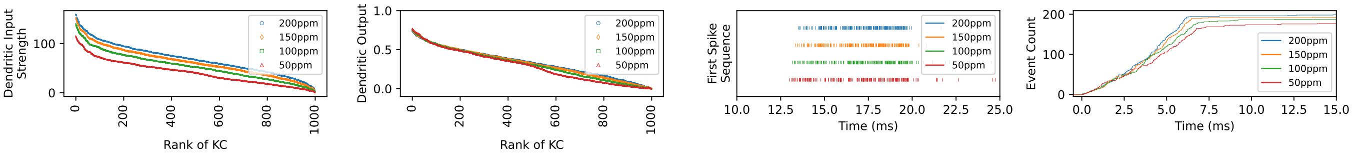

(91) Z3-Hexenol

Figure S6: Continued.

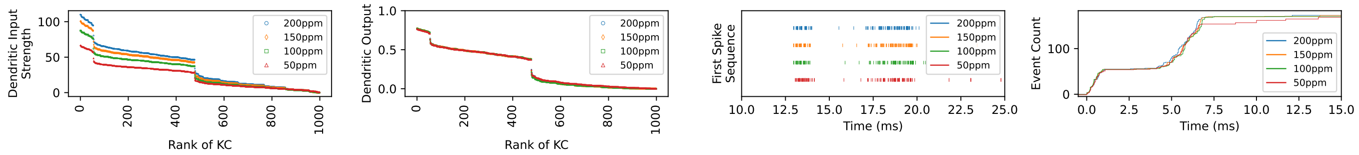

(92) Benzyl Alcohol

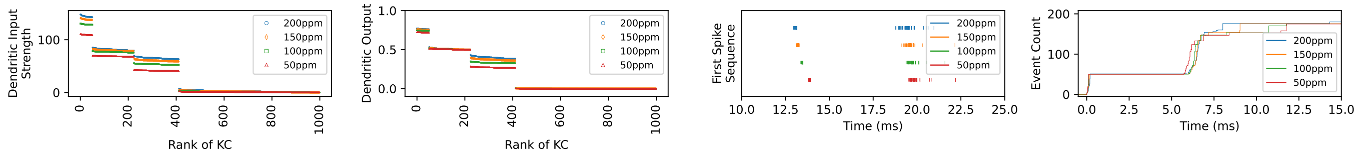

(93) Diethyl Succinate

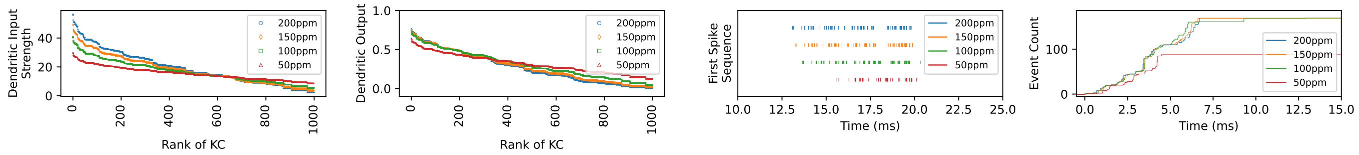

(94) Acetic Acid

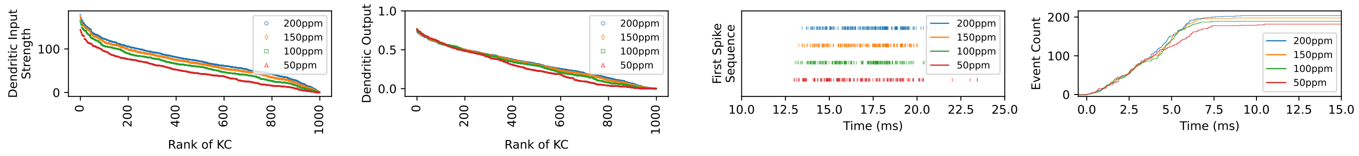

(95) E3-Hexenol

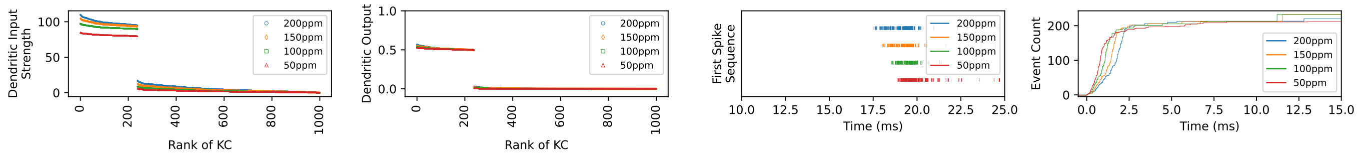

(96) Pentanoic Acid

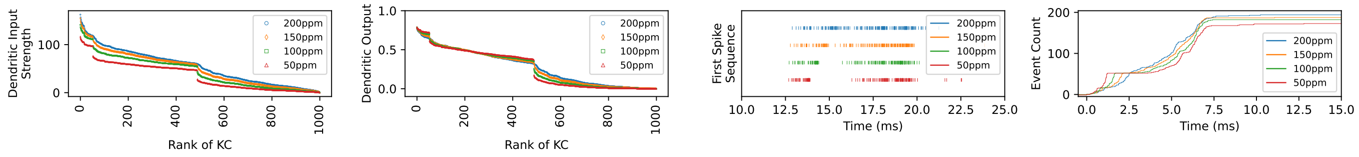

(97) 1-Butanol

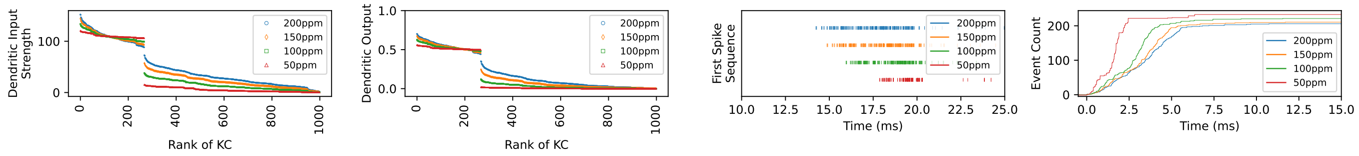

(98) Ethyl Lactate

Figure S6: Continued.

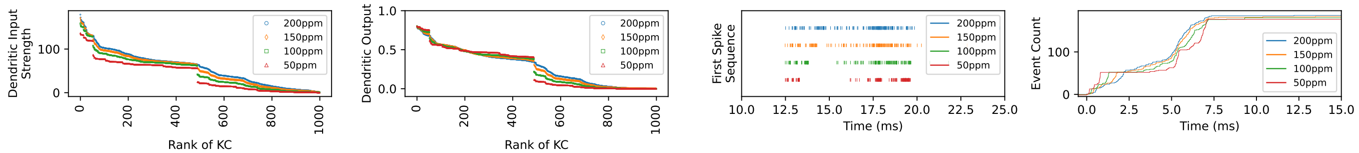

(99) E2-Hexenal

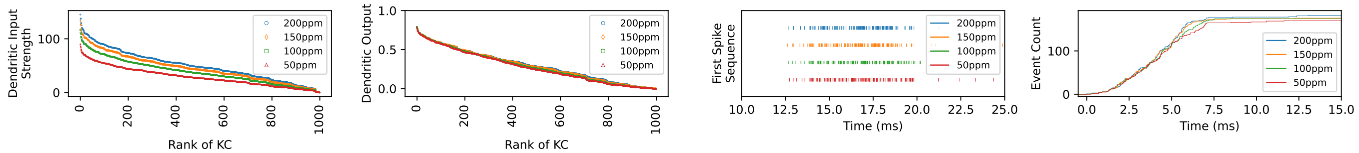

(100) 2-Pentanone

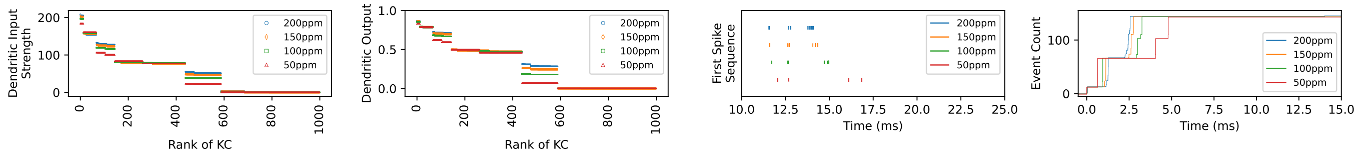

(101) Ethyl Benzoate

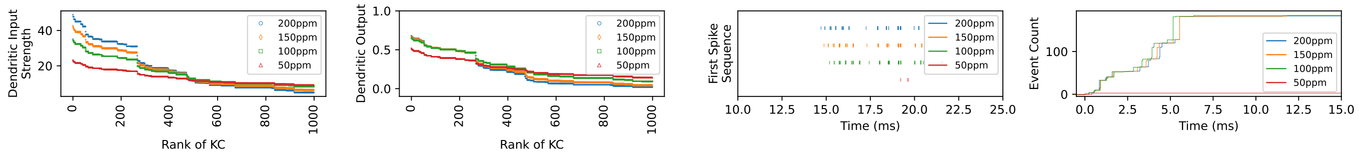

(102) (-)-Trans-caryophyllene

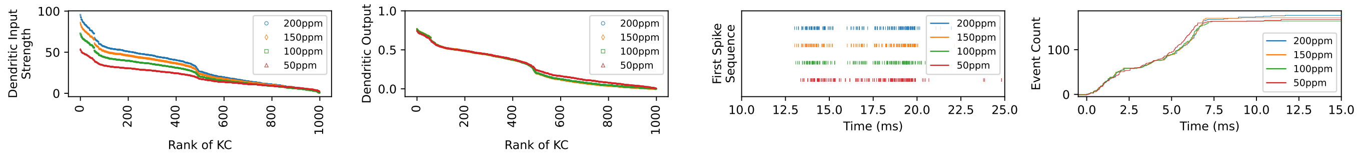

(103) Linalool Oxide

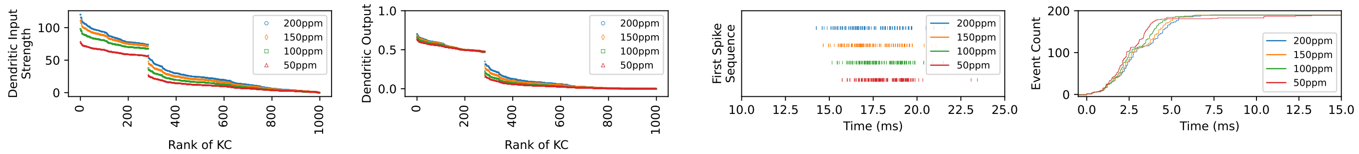

(104) Pentanal

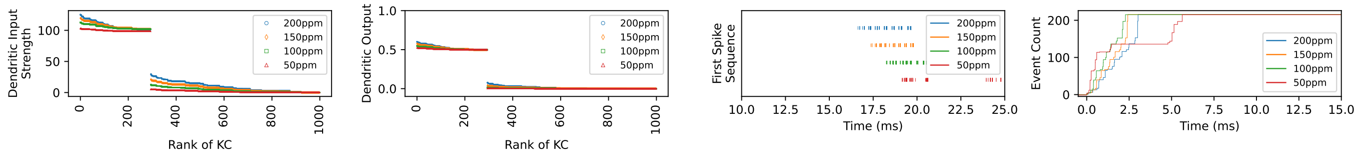

(105) 2-Methylphenol

Figure S6: Continued.

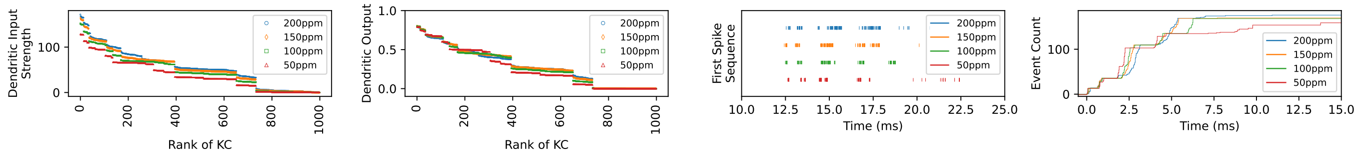

(106) Hexanal

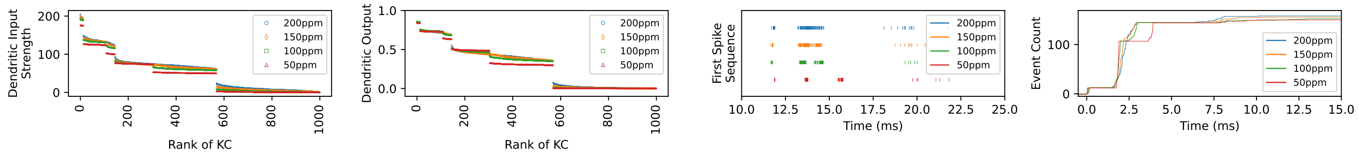

(107) 6-Methyl-5-hepten-2-one

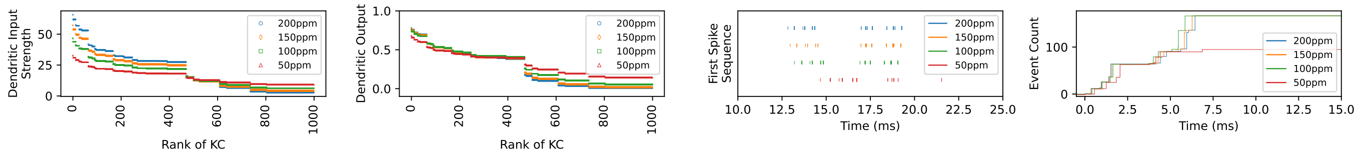

(108) 4-Ethyl Guaiacol

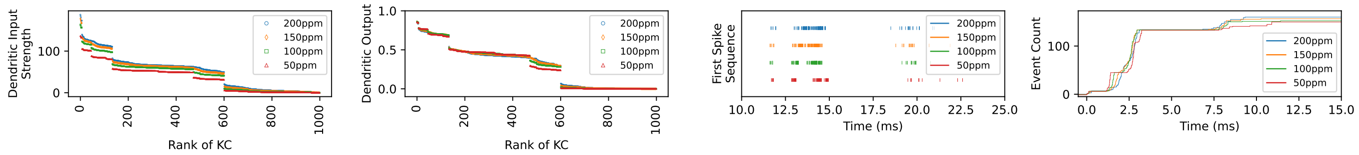

(109) Hexyl Acetate

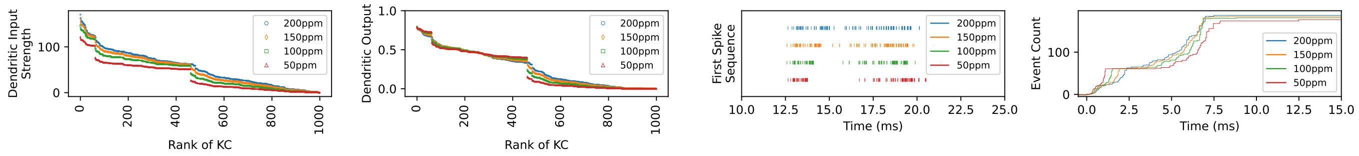

(110) Benzaldehyde

Figure S6: Continued.

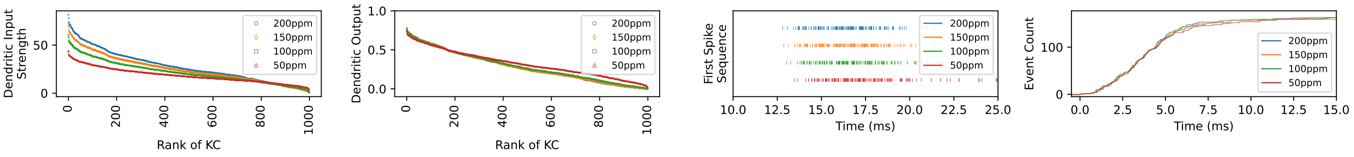

(A) 4:1

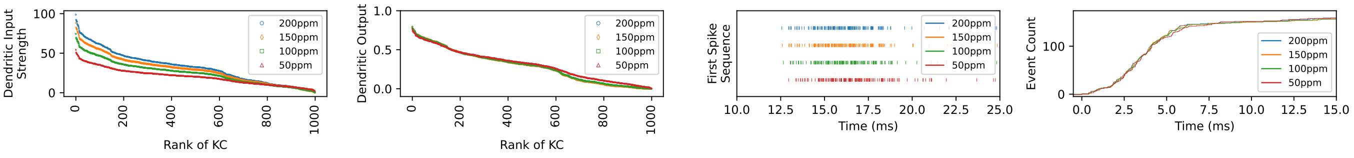

(B) 2:1

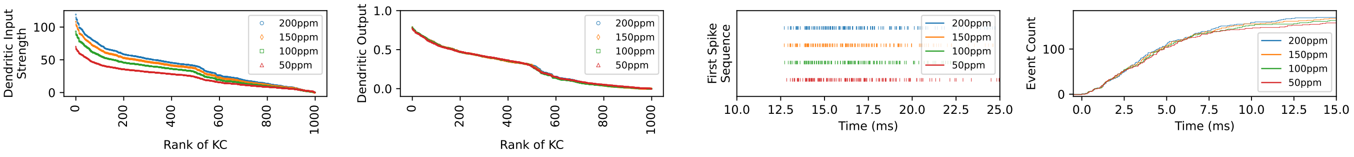

(C) 1:1

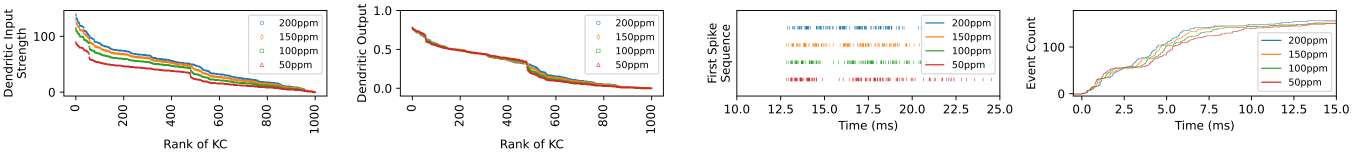

(D) 1:2

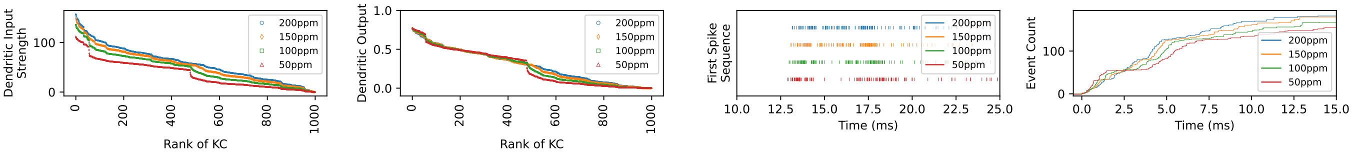

(E) 1:4

Figure S7: Odorant semantics information of a mixture of Methanol and Benzyl Alcohol with different constant concentration amplitude ratios encoded in the time domain across the population of KCs. The mixtures are presented at a fixed ratio in row (**A**) 4:1, (**B**) 2:1, (**C**) 1:1, (**D**) 1:2 and (**E**) 1:4. For each fixed ratio, 4 Methanol concentration levels are used, (red) 50ppm, (green) 100ppm, (orange) 150ppm and (blue) 200ppm. Legend shows this concentration of Methanol. Concentration of Benzyl Alcohol can be derived from the component ratio. **(1st column)** Ranking of KC dendritic inputs. **(2nd column)** Ranking of KC dendritic outputs. **(3rd column)** The first spike sequence code. The first spikes of each of the active KCs in response to each odorant are collected onto a single row for each of the odorant concentration amplitude values **(4th column)** The cumulative interspike intervals of the first spike sequence code in the third column.

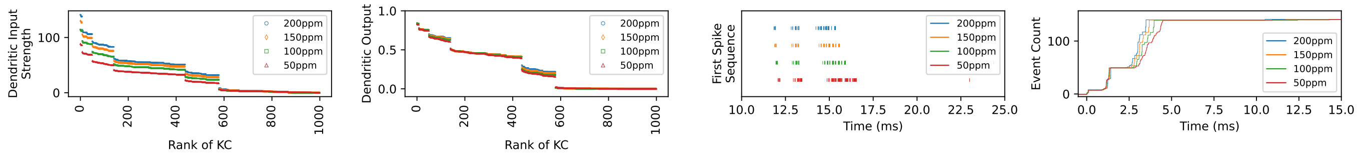

(A) 4:1

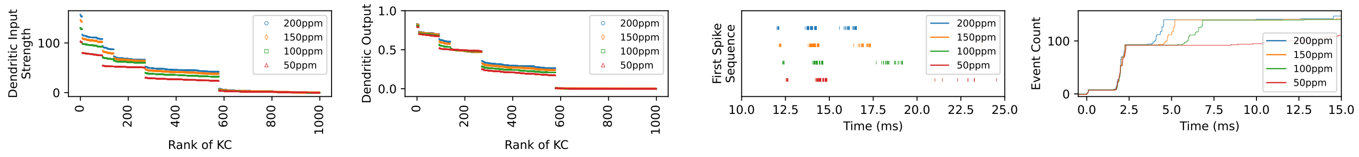

(B) 2:1

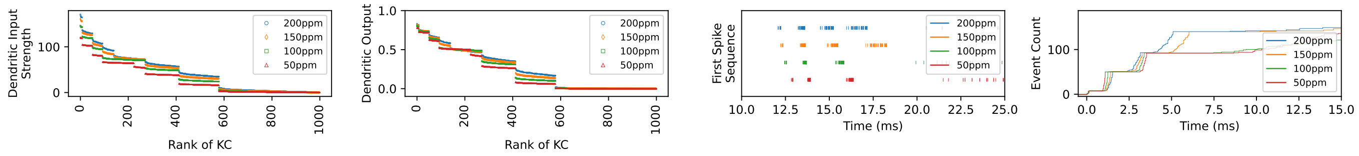

(C) 1:1

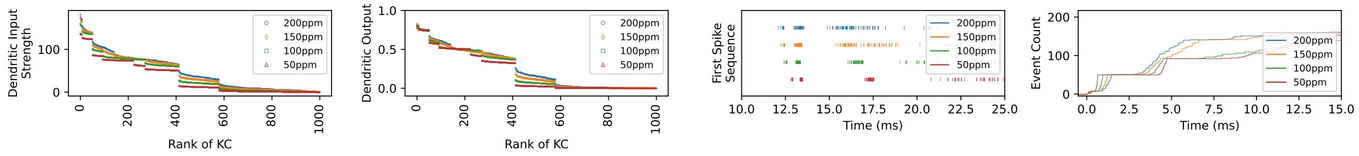

(D) 1:2

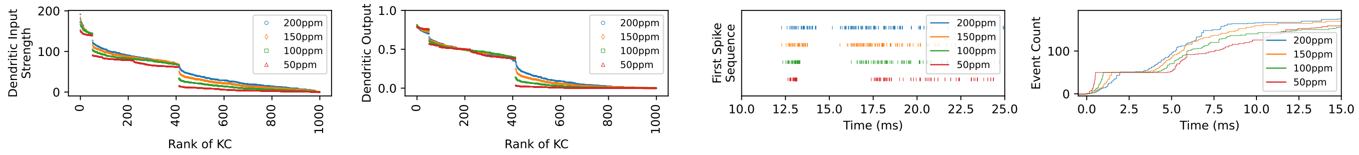

(E) 1:4

Figure S8: Odorant semantics information of a mixture of Acetone and Diethyl Succinate with different constant concentration amplitude ratios encoded in the time domain across the population of KCs. The mixtures are presented at a fixed ratio in row (**A**) 4:1, (**B**) 2:1, (**C**) 1:1, (**D**) 1:2 and (**E**) 1:4. For each fixed ratio, 4 Acetone concentration levels are used, (red) 50ppm, (green) 100ppm, (orange) 150ppm and (blue) 200ppm. Legend shows this concentration of Acetone. Concentration of Diethyl Succinate can be derived from the component ratio. **(1st column)** Ranking of KC dendritic inputs. **(2nd column)** Ranking of KC dendritic outputs. **(3rd column)** The first spike sequence code. The first spikes of each of the active KCs in response to each odorant are collected onto a single row for each of the odorant concentration amplitude values **(4th column)** The cumulative interspike intervals of the first spike sequence code in the third column.

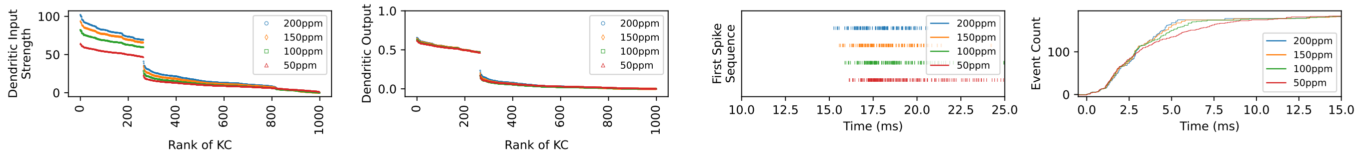

(A) 4:1

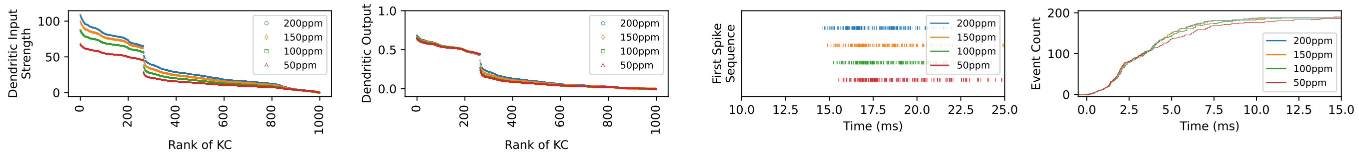

(B) 2:1

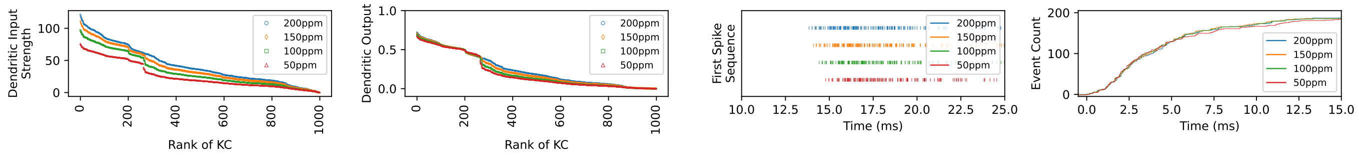

(C) 1:1

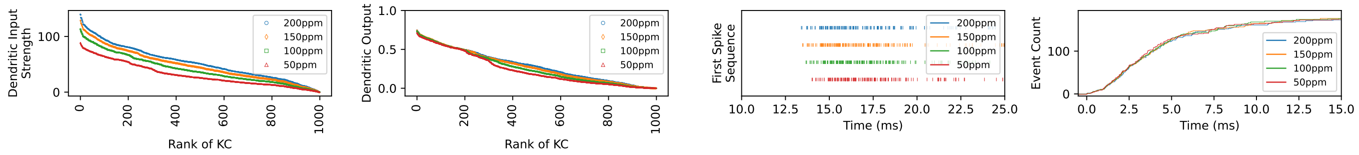

(D) 1:2

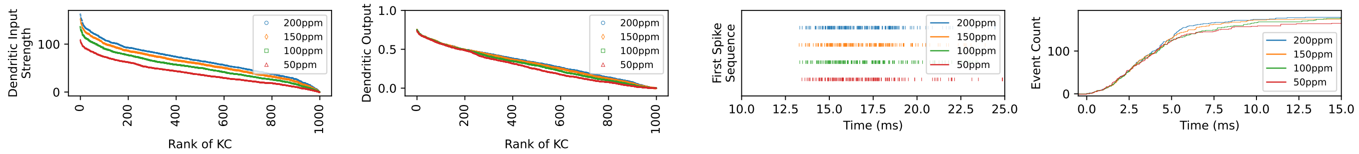

(E) 1:4

Figure S9: Odorant semantics information of a mixture of Acetone and 2,3-Butanedione with different constant concentration amplitude ratios encoded in the time domain across the population of KCs. The mixtures are presented at a fixed ratio in row (**A**) 4:1, (**B**) 2:1, (**C**) 1:1, (**D**) 1:2 and (**E**) 1:4. For each fixed ratio, 4 Acetone concentration levels are used, (red) 50ppm, (green) 100ppm, (orange) 150ppm and (blue) 200ppm. Legend shows this concentration of Acetone. Concentration of 2,3-Butanedione can be derived from the component ratio. **(1st column)** Ranking of KC dendritic inputs. **(2nd column)** Ranking of KC dendritic outputs. **(3rd column)** The first spike sequence code. The first spikes of each of the active KCs in response to each odorant are collected onto a single row for each of the odorant concentration amplitude values **(4th column)** The cumulative interspike intervals of the first spike sequence code in the third column.
